# Is Economic Growth Associated with Reduction in Child Undernutrition in India?

**DOI:** 10.1371/journal.pmed.1000424

**Published:** 2011-03-08

**Authors:** Malavika A. Subramanyam, Ichiro Kawachi, Lisa F. Berkman, S. V. Subramanian

**Affiliations:** 1 Center for Integrative Approaches to Health Disparities, School of Public Health, University of Michigan, Ann Arbor, Michigan, United States of America; 2 Department of Society, Human Development and Health, Harvard School of Public Health, Boston, Massachusetts, United States of America; 3 Harvard Center for Population and Development Studies, Cambridge, Massachusetts, United States of America; Umeå Centre for Global Health Research, Sweden

## Abstract

**Background:**

Economic growth is widely perceived as a major policy instrument in reducing childhood undernutrition in India. We assessed the association between changes in state per capita income and the risk of undernutrition among children in India.

**Methods and Findings:**

Data for this analysis came from three cross-sectional waves of the National Family Health Survey (NFHS) conducted in 1992–93, 1998–99, and 2005–06 in India. The sample sizes in the three waves were 33,816, 30,383, and 28,876 children, respectively. After excluding observations missing on the child anthropometric measures and the independent variables included in the study, the analytic sample size was 28,066, 26,121, and 23,139, respectively, with a pooled sample size of 77,326 children. The proportion of missing data was 12%–20%. The outcomes were underweight, stunting, and wasting, defined as more than two standard deviations below the World Health Organization–determined median scores by age and gender. We also examined severe underweight, severe stunting, and severe wasting. The main exposure of interest was per capita income at the state level at each survey period measured as per capita net state domestic product measured in 2008 prices. We estimated fixed and random effects logistic models that accounted for the clustering of the data. In models that did not account for survey-period effects, there appeared to be an inverse association between state economic growth and risk of undernutrition among children. However, in models accounting for data structure related to repeated cross-sectional design through survey period effects, state economic growth was not associated with the risk of underweight (OR 1.01, 95% CI 0.98, 1.04), stunting (OR 1.02, 95% CI 0.99, 1.05), and wasting (OR 0.99, 95% CI 0.96, 1.02). Adjustment for demographic and socioeconomic covariates did not alter these estimates. Similar patterns were observed for severe undernutrition outcomes.

**Conclusions:**

We failed to find consistent evidence that economic growth leads to reduction in childhood undernutrition in India. Direct investments in appropriate health interventions may be necessary to reduce childhood undernutrition in India.

*Please see later in the article for the Editors' Summary*

## Abstract 2

An analysis of cross-sectional data from repeated household surveys in India, combined with data on economic growth, fails to find strong evidence that recent economic growth in India is associated with a reduction in child undernutrition.

## Editors' Summary

**Background:**

Good nutrition during childhood is essential for health and survival. Undernourished children are more susceptible to infections and more likely to die from common ailments such as diarrhea than well-nourished children. Thus, globally, undernutrition contributes to more than a third of deaths among children under 5 years old. Experts use three physical measurements to determine whether a child is undernourished. An "underweight" child has a low weight for his or her age and gender when compared to the World Health Organization Child Growth Standards, which chart the growth of a reference population. A "stunted" child has a low height for his or her age; stunting is an indicator of chronic undernutrition. A "wasted" child has a low weight for his or her height; wasting is an indicator of acute undernutrition and often follows an earthquake, flood, or other emergency. The prevalence (how often a condition occurs within a population) of undernutrition is particularly high in India. Here, almost half of children under the age of 3 are underweight, about half are stunted, and a quarter are wasted.

**Why Was This Study Done?:**

Although the prevalence of undernutrition in India is decreasing, progress is slow. Economic growth is widely regarded as the major way to reduce child undernutrition in India. Economic growth, the argument goes, will increase incomes, reduce poverty, and increase access to health services and nutrition. But some experts believe that better education for women and reduced household sizes might have a greater influence on child undernutrition than economic growth. And others believe that healthier, better fed populations lead to increased economic growth rather than the other way around. In this study, the researchers assess the association between economic growth and child undernutrition in India by analyzing the relationship between changes in per capita income in individual Indian states and the individual risk of undernutrition among children in India.

**What Did the Researchers Do and Find?:**

For their analyses, the researchers used data on 77,326 Indian children that were collected in the 1992–93, 1998–99, and 2005–06 National Family Health Surveys; these surveys are part of the Demographic and Health Surveys, a project that collects health data in developing countries to aid health-system development. The researchers used eight "ecological" statistical models to investigate whether there was an association between underweight, stunting, or wasting and per capita income at the state level in each survey period; these ecological models assumed that the risk of undernutrition was the same for every child in a state. They also used 10 "multilevel" models to quantify the association between state-level growth and the individual-level risk of undernutrition. The multilevel models also took account of various combinations of additional factors likely to affect undernutrition (for example, mother's education and marital status). In five of the ecological models, there was no statistically significant association between state economic growth and average levels of child undernutrition at the state level (statistically significant associations are unlikely to have arisen by chance). Similarly, in eight of the multilevel models, there was no statistical evidence for an association between economic growth and undernutrition.

**What Do These Findings Mean?:**

These findings provide little statistical support for the widely held assumption that there is an association between the risk of child undernutrition and economic growth in India. By contrast, a previous study that used data from 63 countries collected over 26 years did find evidence that national economic growth was inversely associated with the risk of child undernutrition. However, this study was an ecological study and did not, therefore, allow for the possibility that the risk of undernutrition might vary between children in one state and between states. Further, the target of inference in this study was "explaining" between-country differences, while the target of inference in this analysis was explaining within country differences over time. The researchers suggest several reasons why there might not be a clear association between economic growth and undernutrition in India. For example, they suggest, economic growth in India might have only benefitted privileged sections of society. Whether this or an alternative explanation accounts for the lack of an association, it seems likely that further reductions in the prevalence of child undernutrition in India (and possibly in other developing countries) will require direct investment in health and health-related programs; expecting economic growth to improve child undernutrition might not be a viable option after all.

**Additional Information:**

Please access these websites via the online version of this summary at http://dx.doi.org/10.1371/journal.pmed.1000424.

- The charity UNICEF, which protects the rights of children and young people around the world, provides detailed statistics on child undernutrition and on child nutrition and undernutrition in India
- The WHO Child Growth Standards are available (in several languages)
- More information on the Demographic and Health Surveys and on the Indian National Family Health Surveys is available
- The United Nations Millennium Development Goals website provides information on ongoing world efforts to reduce hunger and child mortality

## Introduction

**Figure 1:**
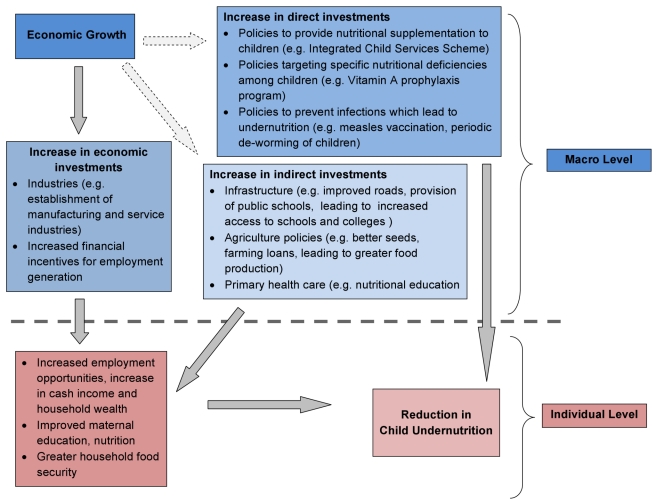
Pathways connecting state-level economic growth and child undernutrition.

Macro-economic growth is considered a major, and often the only, policy instrument to improving health and nutrition in developing countries [1]–[3]. The premise is that economic growth will improve incomes, especially among the poor, and increase their access to and consumption of health-promoting goods and services, leading to improved nutritional status. This argument has also been made in the context of reducing undernutrition in developing countries (Figure 1) [4]. One can postulate three non-exclusive pathways through which economic growth could improve nutritional status among children. These include (i) an increase in income for all, (ii) reduction in poverty, and (iii) investment in public programs, such as the Integrated Child Development Services Scheme, which directly or indirectly could lead to improvement in children's nutritional status [3],[5]. The distinction between pathways related to “reduction in poverty” and “increases in income for all” is important as it emphasizes the importance of income increases among the poor. One might, for instance, expect the effect of income on nutrition to be considerably stronger among those with low incomes, as opposed to income increases at the higher end of the distribution, where further increases might not result in proportionally higher nutritional dividends. While the first two pathways rely on behavioral change at the individual or household level as a result of improved economic standard of living, the third underscores the role of public investment facilitated either by greater economic growth and potential increases in revenue or independent of it [6],[7]. The success of such a “growth-mediated” strategy to reducing undernutrition is, however, neither automatic nor necessary [8]–[10]. For instance, factors such as education of women and household size have been shown to have a greater influence on the nutrition of a household than macro-economic growth translated to improvements in income leading to improvements in nutritional outcomes [11],[12]. Further, there is a body of research arguing that it is healthier populations, for example with healthy nutritional indicators, that are a pre-requisite for increased economic growth and improved standard of living [13],[14].

To our knowledge only one previous study has focused on the relationship between economic growth and child undernutrition [3]. This study used data from 63 countries and found that economic growth at the national level was inversely associated with risk of child undernutrition. However, this study was an ecological analysis and used data from 1970 to 1996. Ecological analyses assume that the risk of undernutrition is the same for every child within a state. The biggest shortcoming of ecological models is their inability to quantify the association between *country-level* growth and *individual-level* risk of undernutrition, which inherently is a multilevel question. Over the last two decades India has experienced rapid economic growth, with growth rates greater than 7% between 1994 and 1997 and a 9% or greater rate after 2005 [15]. The persistence of undernutrition among children in India also remains a critical public health concern [16]–[18]. We are not aware of any study that has applied a multilevel framework and used recent data to empirically examine whether improvements in economic growth have led to reductions in the risk of undernutrition among children in India.

Using three waves of nationally representative micro-data on childhood undernutrition as well as macro-data on state economic growth, we investigated the association between economic growth and child undernutrition in India.

## Methods

### Data

Data for this study came from three rounds of the NFHS conducted in India in 1992–93, 1998–99, and 2005–06. These repeated cross-sectional surveys were established to especially collect data on maternal and child health indicators that are representative at the national and state levels [19]. The NFHS is part of the Demographic and Health Surveys (DHS) that are operational in more than 80 countries (http://www.measuredhs.com/aboutsurveys/dhs/start.cfm).

### Sampling Plan

The NFHS used a multi-stage stratified cluster sampling design to collect data from respondents across India [19],[20]. At the first stage, populations were stratified by urban and rural area of residence in each state. The sample size at the state level was proportional to the size of the state's urban and rural populations. In rural areas villages or clusters of villages were the primary sampling units and they were selected based on a probability proportional to population size (PPS), followed by a random selection of households within villages. The urban sample was obtained by selecting wards with PPS, followed by a random selection of one census enumeration block within the sample ward, and then a random selection of households. Within the urban and rural households, all ever-married women aged 15–49 years who resided the previous night in the household were qualified to be respondents in the survey. All three surveys largely followed the above sampling scheme, with a few differences. For instance, in 1992, the urban sample was selected by first stratifying urban areas into (i) extremely large cities, (ii) district headquarters, and (iii) other towns, followed by random selection of census enumeration blocks within them, and then by random selection of households within the blocks. In the same survey, the age range for eligibility was 13–49 years as compared to 15–49 in other rounds. Meanwhile, in 2005, the ever-married criterion was not applied as an eligibility condition. The response rate for women was 96.1% in 1992–93, 95.5% in 1998–99, and 94.5% in 2005–06 [20].

### Study Population and Sample Size

**Figure 2:**
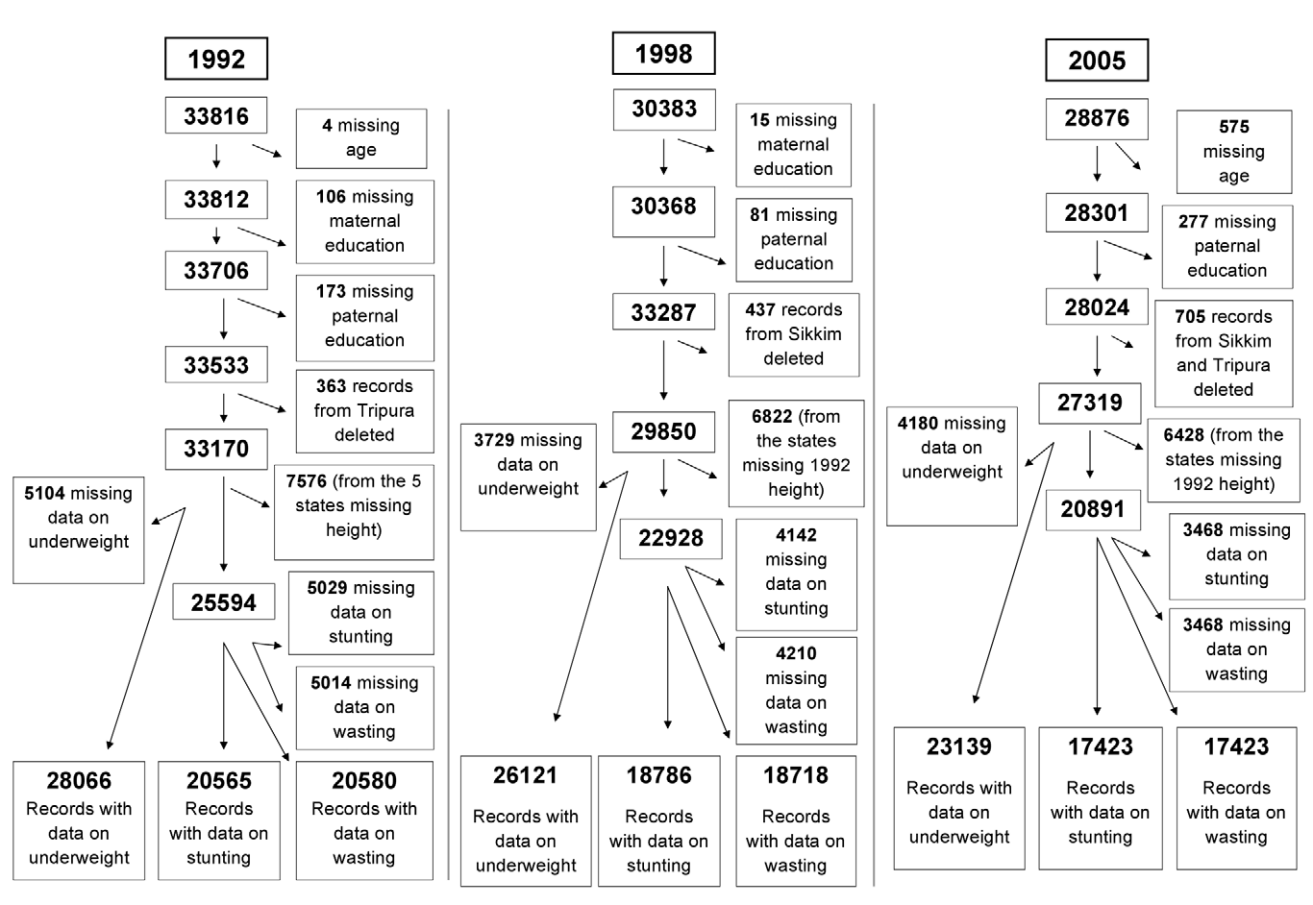
Scheme of application of exclusion criteria to data from the three rounds of Indian NFHS surveys (1992–93, 1998–99, and 2005–06).

In order to ensure comparability of data across different waves, we applied the following inclusion criteria; we only used data on children born as singletons to ever-married women aged 15–49 who participated in the interview in any of the three surveys, and were between 0 and 35 months in age and alive at the time of survey (*n* = 93,075). We excluded children missing data on height or weight (*n* = 13,013 for underweight, *n* = 12,639 for stunting, and *n* = 12,692 for wasting) and covariates such as age and parental education (*n* = 1,231). Information on children was not ascertained in the states of Sikkim in 1992–93 and Tripura in 1998–99. In order to make the samples comparable across surveys, we excluded 1,505 records from these two states in the years that the data were available. Figure 2 shows the original sample size for each of the three survey waves, details of inclusion and exclusion criteria, and the resulting analytic sample size for each survey. In the 1992–93 wave, there were 33,816 children who met the inclusion criteria. We excluded children with missing data on covariates (*n* = 597) and those residing in Tripura (*n* = 363). We further excluded those with missing data on weight (*n* = 5,104), with a sample size of 28,066 children (83% of the 33,816 eligible children) for the underweight analysis. For the analyses related to stunting and wasting, we had to exclude 7,576 children as height was not measured in the states of Andhra Pradesh, Himachal Pradesh, Madhya Pradesh, Tamil Nadu, and West Bengal in the 1992–93 wave. Additionally, excluding those missing data on height (*n* = 5,029) and height and weight (*n* = 5,014) gave us the final analytic samples of 20,565 (78.40% of the 26,230 eligibles) and 20,580 (78.46% of the 26,230 eligibles) for the analyses of stunting and wasting, respectively.

In the 1998–99 survey, 30,383 children met the inclusion criteria. Excluding children missing data on covariates (*n* = 96) and those residing in Sikkim (*n* = 437), there were 29,850 observations. Further excluding those missing data on weight (*n* = 3,729) yielded 26,121 (85.97% of the 30,383 eligibles) observations for the underweight analysis. Additionally, we excluded 6,822 children from the stunting and wasting analyses because they were from the five states where height was not measured in 1992. Further excluding those missing data on height (*n* = 4,142) and height and weight (*n* = 4,210) gave us the final analytic samples of 18,786 (79.73% of the 23,561 eligibles) for stunting and 18,718 (79.45% of the 23,561eligibles) for wasting.

Among the 28,876 who met the inclusion criteria in 2005–06, we excluded children with missing data on covariates (*n* = 852) and those residing in Sikkim or Tripura (*n* = 705). Excluding those missing data on weight (*n* = 4,180) resulted in an analytic sample of 23,139 observations (80.13% of the 28,876 eligibles) for the underweight analysis. Also, 6,428 children, from the states where height was not measured in 1992, were excluded from the stunting and wasting analyses. The same number of observations (*n* = 3,468) were missing data for height as well as height and weight, resulting in a final analytic sample of 17,423 (77.62% of the 22,448 eligibles) for both stunting and wasting.

We then pooled data from the 1992–93, 1998–99, and 2005–06 surveys for a final sample size of 77,326, 56,774, and 56,721 for the analyses of underweight, stunting, and wasting, respectively. Three new states were created between 1992 and 2006: Bihar had been split into Chhattisgarh and Bihar, Madhya Pradesh split into Jharkhand and Madhya Pradesh, and Uttar Pradesh split into Uttaranchal and Uttar Pradesh. To handle the issue of new states, we pooled the information of the new states with the data from their parent states.

### Outcomes

We defined children's undernutrition status based on their anthropometry along three dimensions: weight-for-age, height-for-age, and weight-for-height [21],[22]. Weight of the child was obtained by trained investigators who weighed each child with a solar-powered scale accurate to within 100 g. The investigators also measured each child's height with an adjustable measuring board calibrated in millimeters [23]. We used the internationally accepted World Health Organization Child Growth Standards to classify a child as undernourished or not. Specifically, we applied the “standard” version of the SAS macro provided by WHO (http://www.who.int/childgrowth/software/en/) in order to calculate the *z* scores for each child's weight-for-age, height-for-age, and weight-for-height. The SAS macro involves a lengthy computation that accomplishes the equivalent of dividing a child's weight by the median weight for a child of that age and sex, dividing a child's height by the median height for a child of that age and sex, and dividing a child's weight by the median weight for a child of that height and sex. Each of these numbers is then standardized as a *z* score with a mean of 0 and a standard deviation (SD) of 1. Each anthropometric measure of undernutrition was defined as “present” if the *z* score was more than 2 SDs below the World Health Organization–determined median scores [24]. These median values differ by age (measured in months) and gender, are considered the international standard, and have been computed by the WHO Multicentre Growth Reference Study. Thus, children whose weight-for-age *z* scores were more than 2 SDs below the median for their age and gender were defined as being underweight, those with height-for-age *z* scores more than 2 SDs below the median were defined as being stunted, and those with weight-for-height *z* scores more than 2 SDs below the median were defined as wasted. We also considered severe anthropometric failure, defined as more than 3 SDs below the World Health Organization–determined median scores. Thus, children whose weight-for-age *z* scores were more than 3 SDs below the median for their age and gender were defined as being severely underweight, those with height-for-age *z* scores more than 3 SDs below the median were defined as being severely stunted, and those with weight-for-height *z* scores more than 3 SDs below the median were defined as severely wasted [24].

### Exposure

We used per capita net state domestic product (hereafter referred to as state per capita income), expressed in Indian Rupees (INR), as a measure of a state's economic development. These data were obtained from the Reserve Bank of India, for the years 1993, 1998, and 2005, and measured in 2008 rupees [25]. The state per capita income is a measure of the economic performance of a state, with higher values indicating higher levels of economic development. Formally, it is the value of all goods and services produced within the boundaries of a state for that year, minus the cost of capital used in the production. Our measure of state per capita income correlates highly with estimates from other independent surveys [26]. Further, this measure is used by the Government of India to allocate central resources among various states [27]. Since per capita income was measured at multiple times for the same state, by including it in regression models that account for state effects, we were able to estimate the effect of “within” state change in per capita income, which essentially can be interpreted as economic growth. To overcome the issue of new states that were created after 1992, we imputed the mean of the state per capita income of the new states and that of their parent states as the state per capita income of the parent state, ensuring repeated measures of state per capita income for every state for each of the three waves. In the analysis, state per capita income was centered at its mean and divided by 5,000 to get estimates in units of 5,000 INR (∼$107). We also computed a “percent change in per capita income” measure for use in sensitivity analyses. The measure was calculated as follows: per capita income in 2005 minus per capita income in 1998 divided by per capita income in 1998, expressed as a percentage. Similar “percent change” measures were computed using differences between per capita incomes in 1998 and 1992 as well as 2005 and 1992.

### Covariates

Age, sex, and birth order of the child; mother's age, marital status, and education; father's education, caste, and religion; and household wealth, urban/rural status, survey year, and state of residence were included as covariates in the study (Table S1). Age was measured in months and centered around 18, the mean. Birth order was categorized as first, second, third, fourth, and fifth or greater. Mother's age in years was classified as less than 17, 17–19, 20–24, 25–29, and more than 29 years. Mother's marital status at the time of survey was classified as married if she was living with her husband and as unmarried if she was widowed, divorced, or separated. Mother's and father's education were defined using years of schooling and grouped using important benchmarks in the Indian educational system: 0 (no schooling), 1–5 (primary), 6–10 (secondary), 11–12 (higher secondary), and 13 or more (some college or more). Caste identification was based on the self-reports of the mother and was grouped as scheduled caste, scheduled tribe, general caste, or no caste. Scheduled castes are those whose members have the greatest burden of deprivation within the caste system [28]. Scheduled tribes include approximately 700 officially recognized social groups that have historically been geographically and socially isolated and represent the “indigenous” groups in India [29]. The general caste is a residual category containing those not identifying themselves as members of legislatively recognized marginalized classes of scheduled castes or tribes, but includes the “other backward class” and the “high”-caste groups. “Other backward class” is a legislatively defined group representing those who have historically been subject to significant deprivation that is not as severe as that of scheduled castes and tribes. “High” caste groups are those groups of castes that are historically and socially considered to be at the top of the caste-based hierarchy. Religion of the child was based on the head of household's self-identification as Hindu, Muslim, Christian, Sikh, or other/missing religion. Household wealth was measured using an asset index, defined in terms of ownership of material possessions [30], with each household assigned a wealth score based on a combination of different household characteristics that were weighted according to a factor analysis procedure. For this procedure, *z* scores were calculated for each indicator variable and a principal components analysis was performed using these *z* scores. For each household, the values of the indicator variables were multiplied by the factor loadings of the first principal component and summed to produce a standardized household index value with a mean of 0 and a standard deviation of 1. This standardized score was then divided into quintiles. In order to capture the relative disparity among different wealth quintiles in each year, the quintiles were created separately for each survey. Using the 2001 Indian National Census definition, households were grouped based on location in either an urban area or a rural village. Survey year was included using one indicator variable each for the three surveys. Similarly, indicator variables for each state were used in fixed effects models.

### Analysis

We estimated multilevel logistic regression models with a log link function to analyze the binary outcomes associated with underweight, stunting, and wasting, which in turn provided the odds ratios (ORs) along with their 95% confidence intervals. We accounted for the clustering in our observations due to mothers and primary sampling unit by specifying a random effect for each mother and primary sampling unit. We estimated our models with states specified as random effects (thus accounting for non-independence among observations within a state) and as fixed effects in separate models; while the former has the advantage of being more efficient [31], the latter is often considered to be less biased as all the observed and unobserved characteristics of the state that are time-constant are accounted for [32]. We conducted our analysis with both approaches also as a test of sensitivity of our findings to choice of modeling strategy. As is required in models with repeated cross-sectional measurements, as well as to account for the survey period differences, indicator variables for survey year 1998–99 and 2005–06 were included, with the reference being survey period 1992–93. Adjusting for the survey year also accounts for any national-level unique changes occurring during that year that might affect child undernutrition and also be associated with state-level economic growth. We did not specify state-specific survey-period differences in order to not control for factors that could have been due to state economic growth and resulted in reduction of child undernutrition. Model estimation was based on penalized quasi-likelihood procedures with first-order Taylor linearization as implemented in MLwiN [33]. Data management was performed in SAS [34].

We first estimated a model with only per capita state income that simply accounted for the repeated cross-sectional data structure. We then added child's age and gender in the second model, and then added birth order, maternal age, maternal education, paternal education, household wealth, caste, religion, and urban residence (fully adjusted model). We repeated these models also for severe undernutrition measures. To test how robust our findings were to choice of model, we fit a series of ecological and multilevel models using the per capita income measure in 5,000 INR units as well as a measure of percentage change in per capita income. We additionally conducted sensitivity analyses to determine if the missing data were differentially distributed across covariates and if the proportion of missing data in a state was related to its per capita income. The sensitivity analyses also included fitting a second set of multilevel models for stunting and wasting using data only from 1998 and 2005, in order to include data from the five states that were missing height data in 1992.

### Research Ethics

The NFHS has been conducted under the scientific and administrative supervision of the International Institute for Population Sciences, Mumbai, India, a regional center for teaching, training, and research in population studies that is associated with the Ministry of Health and Family Welfare of the Government of India. The institute conducts an independent ethics review of the NFHS protocol. Data collection procedures were also approved by the ORC Macro (Calverton, Maryland) institutional review board. Oral informed consent for the interview/survey and measurements was obtained by interviewers from the participating mothers [20]. The present analysis was reviewed by the Harvard School of Public Health Institutional Review Board and was considered exempt from full review because the study was based on an anonymous public use data set with no identifiable information on the survey participants.

## Results

The distribution of covariates did not differ in any substantial manner between children with and without missing data on the outcomes. The greatest difference between records with and without missing data on underweight (Table S2) was in the distribution of maternal education (*p*<0.001) in 1998–99, where 65.59% of those missing data had zero years of education versus 52.28% among those without missing data. The distribution of maternal education between those with and without missing data showed the greatest difference for stunting and wasting as well (*p*<0.001 for both outcomes). While the difference was 67.36% versus 54.61% for stunting (Table S3), it was 66.45% versus 54.81% for wasting (Table S4). There was variation in the proportion of missing data across states, ranging from 4.7% to 41.89% in 1992–93, 3.23% to 37.13% in 1998–99, and 5.7% to 42.28% in 2004–05. However, there was no correlation (substantively and statistically) between the proportion of missing data in a state and its per capita income (Table S5). The proportion of missing data was negatively correlated with state per capita income in 1992–93 and 1998–99; for example, the correlations for stunting were *r* = −0.37, *p* = 0.12 in 1992–93, and *r* = −0.27, *p* = 0.26 in 1998–99. However, the correlation was positive in 2004–05 (for stunting, *r* = 0.39, *p* = 0.10).

**Figure 3:**
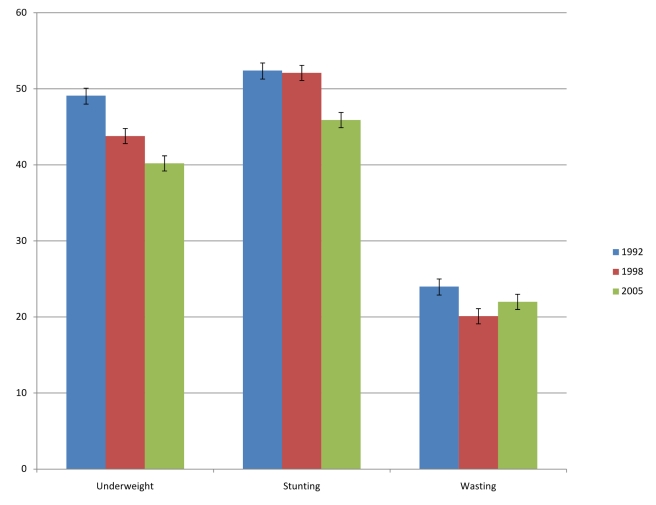
Weighted prevalence (%) of underweight, stunting, and wasting in 1992, 1998, and 2005. Error bars are 95% confidence intervals.

The prevalence of underweight decreased from 49.1% (CI 48.1%–50.2%) in 1992–93 to 43.8% (CI 42.9%–44.8%) in 1998–99 to 40.2% (CI 39.1%–41.3%) in 2005–06 (Figure 3). Stunting prevalence also decreased from 52.4% (CI 51.3%–53.6%) to 45.9% (CI 44.8%–47.1%) during the same time period, while the prevalence of wasting decreased only marginally from 24% (CI 23%–25%) in 1992–93 to 22% (CI 21%–23%) in 2005–06. There was substantial variation between states in each of the measures of undernutrition (Table 1). For instance, in 1992, the prevalence of underweight varied between 19.3% in Mizoram and 60.7% in Bihar, while in 2005–06 it varied between 14.3% in Mizoram and 55.2% in Madhya Pradesh. Other measures of undernutrition also showed substantial state variability over time. The state per capita income increased, on average, from INR 7,965.46 (∼$166) in 1993 to 18,089.50 (∼$377) in 1998 and 26,308.33 (∼$548) in 2005, an increase of 230% over a period of 12 years (Table 2). There was substantial variation both in the levels of economic development as well as the rate of increase during the study period. Bihar was the state with the lowest state per capita income (and rate of growth) at all three time points, while the state with the highest state per capita income was New Delhi in 1993 and Goa in 1999 and 2005 (Table 2). The state that experienced the most growth was Goa. The decline in prevalence of underweight between 1992 and 2005 was 0.46 in Bihar, 0.83 in New Delhi, and 0.74 in Goa (measured in percentage points per year).

**Table 1:** Weighted prevalence (%) of, and rate of change in, underweight, stunting, and wasting, with correlations with economic growth, among children under age 3, for the states of India in 1992–93, 1998–99, and 2005–06.

	Underweight (*N* = 77,326)				Stunting (*N* = 56,774)				Wasting (*N* = 56,721)				
State	1992	1998	2005	Change*	1992	1998	2005	Change*	1992	1998	2005	Change*	Economic Growth*
Andhra Pradesh	42.12	35.00	29.44	−0.98									1,395.75
Arunachal Pradesh	33.12	21.69	29.54	−0.28	55.85	29.65	36.96	−1.45	15.81	10.40	16.86	0.08	1,140.75
Assam	44.73	38.35	35.68	−0.70	55.95	55.83	40.89	−1.16	14.61	18.62	16.63	0.16	957.75
Bihar	60.71	54.25	54.70	−0.46	59.56	56.69	49.25	−0.79	31.68	25.75	33.18	0.12	651.92
Goa	30.84	20.80	21.17	−0.74	35.53	22.17	25.72	−0.75	17.49	15.98	13.28	−0.32	4,233.58
Gujarat	44.03	42.92	40.91	−0.24	50.67	51.20	48.91	−0.14	23.66	20.59	19.74	−0.30	1,701.17
Haryana	31.18	31. 98	38.14	0.54	49.71	55.92	43.22	−0.50	8.04	7.60	22.29	1.10	2,260.17
Himachal Pradesh	38.51	38. 35	31.16	−0.57									2,020.25
Jammu and Kashmir	37.63	29.84	23.59	−1.08	43.86	44.56	32.38	−0.88	19.42	14.89	18.34	−0.08	1,102.50
Karnataka	47.35	39.57	33.13	−1.09	46.85	42.05	42.19	−0.36	24.11	24.93	19.11	−0.38	1,383.83
Kerala	21.77	19.78	20.64	−0.09	32.67	26.53	26.28	−0.49	13.50	13.19	15.54	0.16	1,778.33
Madhya Pradesh	58.06	53.04	55.22	−0.22									896.33
Maharashtra	47.07	45.80	32.36	−1.13	46.98	47.82	43.91	−0.24	27.97	24.02	16.95	−0.85	1,747.42
Manipur	20.40	20.62	19.63	−0.06	32.15	38.87	28.66	−0.27	10.18	9.38	10.93	0.06	1,038.08
Meghalaya	39.16	30.39	42.50	0.26	53.76	47.40	47.75	−0.46	17.86	14.94	31.17	1.02	1,331.75
Mizoram	19.30	19.27	14.25	−0.39	44.28	40.86	35.17	−0.70	5.15	13.89	9.69	0.35	1,241.67
Nagaland	22.63	21.77	23.91	0.10	32.69	37.35	34.21	0.12	12.92	13.60	15.92	0.23	1,105.67
New Delhi	36.21	31.76	25.48	−0.83	46.98	43.40	43.43	−0.27	15.85	16.47	17.63	0.14	3,141.08
Orissa	50.43	50.36	39.03	−0.88	50.19	49.04	43.59	−0.51	28.12	29.74	23.18	−0.38	970.33
Punjab	40.72	26.25	23.14	−1.35	43.80	43.52	33.90	−0.76	21.00	8.69	9.93	−0.85	1,685.33
Rajasthan	45.47	47.48	36.73	−0.67	45.75	56.99	40.03	−0.44	24.42	16.85	22.37	−0.16	933.33
Tamil Nadu	40.46	31.72	26.04	−1.11									1,590.50
Uttar Pradesh	54.76	48.62	40.86	−1.07	59.00	60.09	51.66	−0.56	23.53	16.49	19.34	−0.32	1,022.00
West Bengal	52.17	45.02	37.54	−1.13									1,356.25
Correlation with economic growth				−0.04 (p = 0.86)				0.11 (*p* = 0.66)				−0.03 (*p* = 0.89)	

Economic growth  =  Slope of change in PCSDP (Indian Rupees) per year, over the 1993–2005 period.

*Change  =  (Prevalence in 2005– prevalence in 1992)/13, in percentage point units.

**Table 2:** State-wise distribution in 1993, 1999, and 2005 of, and rate of change between 1993 and 2005 in, per capita net state domestic product (calculated in 2008 Indian Rupees).

State	1993	1999	2005	Change
Andhra Pradesh	7,006	15,507	23,755	1,395.75
Arunachal Pradesh	8,853	14,107	22,542	1,140.75
Assam	5,520	12,282	17,013	957.75
Bihar	4,657	8,600	12,480	651.92
Goa	15,332	42,296	66,135	4,233.58
Gujarat	9,054	18,864	29,468	1,701.17
Haryana	10,526	23,121	37,648	2,260.17
Himachal Pradesh	6,896	20,806	31,139	2,020.25
Jammu and Kashmir	5,400	13,745	18,630	1,102.50
Karnataka	7,242	16,603	23,848	1,383.83
Kerala	6,524	19,294	27,864	1,778.33
Madhya Pradesh	5,516	12,072	16,272	896.33
Maharashtra	12,010	23,340	32,979	1,747.42
Manipur	5,929	13,260	18,386	1,038.08
Meghalaya	5,934	14,611	21,915	1,331.75
Mizoram	7,517	16,443	22,417	1,241.67
Nagaland	7,730	13,819	20,998	1,105.67
New Delhi	17,522	38,682	55,215	3,141.08
Orissa	4,662	10,567	16,306	970.33
Punjab	12,934	25,611	33,158	1,685.33
Rajasthan	5,315	13,619	16,515	933.33
Tamil Nadu	8,051	19,378	27,137	1,590.50
Uttar Pradesh	4,794	11,695	17,058	1,022.00
West Bengal	6,247	15,826	22,522	1,356.25

Change  =  Slope of change in PCSDP in Indian Rupees per year, over the 1993–2005 period.

In models with random effects for state, primary sampling unit, and mother, adjusted only for survey year, an increase in state per capita income of INR 5,000 over 6.5 years was associated with an OR of 1.01 (95% CI 0.98, 1.04) for underweight, OR of 1.02 (95% CI 0.99, 1.05) for stunting, and an OR of 0.99 (95% CI 0.96, 1.02) for wasting (Table 3). Upon additionally adjusting the model for age and sex of the child, the association between change in the per capita state income and the individual risk of being undernourished remained unaltered, with an OR of 1.01 (95% CI 0.98, 1.04) for underweight, OR of 1.02 (95% CI 0.99, 1.05) for stunting, and an OR of 0.99 (95% CI 0.96, 1.02) for wasting. Further adjustment of demographic and socioeconomic covariates measured at the level of mother, father, and household did not alter the unadjusted estimates—the ORs were 1.03 (95% CI 1.00, 1.06) for underweight, 1.04 (95% CI 1.01, 1.07) for stunting, and 1.00 (95% CI 0.97, 1.04) for wasting. While the above estimates were based on states as random effects, the estimates were identical when states were specified as fixed effects (Table 3).

**Table 3:** Odds ratios (95% confidence intervals) for associations between economic growth (in 5,000 INR over a 6.5-year period) and indicators of undernutrition from multilevel logistic models.

	Underweight		Stunting		Wasting	
	Any	Severe	Any	Severe	Any	Severe
Year adjusted^a^	1.01 (0.98, 1.04)	1.02 (0.98, 1.06)	1.02 (0.99, 1.05)	1.06 (1.02, 1.10)	0.99 (0.96, 1.02)	1.02 (0.97, 1.08)
Year, age and sex adjusted^a^	1.01 (0.98, 1.04)	1.02 (0.98, 1.06)	1.02 (0.99, 1.05)	1.06 (1.02, 1.10)	0.99 (0.96, 1.02)	1.02 (0.97, 1.08)
Fully adjusted^b^	1.03 (1.00, 1.06)	1.05 (1.01, 1.09)	1.04 (1.01, 1.07)	1.08 (1.05, 1.12)	1.00 (0.97, 1.04)	1.03 (0.98, 1.09)
Fully adjusted^c^	1.02 (0.99, 1.05)	1.03 (0.99, 1.07)	1.03 (1.00, 1.06)	1.07 (1.04, 1.11)	1.00 (0.97, 1.04)	1.04 (0.99, 1.10)

All estimates conditional on random effects.

a Additionally adjusted for state as *random* effect.

b Adjusted for state (as *random* effect), survey year, age, sex, birth order, maternal age, marital status, maternal education, paternal education, household wealth, caste, religion, and urban/rural residence.

c Adjusted for state (as *fixed* effect), survey year, age, sex, birth order, maternal age, marital status, maternal education, paternal education, household wealth, caste, religion, and urban/rural residence.

The results for “severe” outcomes were similar to the results for “any” undernutrition outcomes, with the exception of severe stunting (Table 3). A 5,000 INR increase in per capita state income over 6.5 years was associated with a marginally higher risk of severe stunting with an OR of 1.06 (95% CI 1.02, 1.10) in the model adjusted for survey year and an OR of 1.08 (95% CI 1.05, 1.12) in the fully adjusted model.

In five of eight possible ecological models that could be estimated with our data, there was no statistically significant association between state economic growth and mean levels of child undernutrition at the state level (Table 4). Similarly, in 8 of 10 multilevel models, there was no statistical support for an inverse association between per capita income (1998) or economic growth (all years) and undernutrition. Per capita income or economic growth was inversely associated with undernutrition in 3 of the ecological and 2 of 10 multilevel models, especially in a multilevel model that used data from all three surveys but did not account for the survey year (Model 17). Upon including survey year, the inverse association was no longer observed in this model (Model 18).

**Table 4:** Results of ecological and multilevel models examining the association of economic growth or per capita income with underweight.

Model	Design	Outcome	Exposure^a^	Covariates^b^	Data	Measure of Association^c^	Growth Reduces Undernutrition?
1	Ecological	State prevalence of underweight	Level of per capita income	None	Only 1992	−4.75 (3.47)	No
2	Ecological	State prevalence of underweight	Level of per capita income	None	Only 1998	−2.63 (1.39)	No
3	Ecological	State prevalence of underweight	Level of per capita income	None	Only 2005	−1.64 (0.82)	No
4	Ecological	Change in state prevalence of underweight	Economic growth (change in per capita income)	State fixed effects	All three years	−1.68 (0.31)***	Yes
5	Ecological	Change in state prevalence of underweight	Economic growth (change in per capita income)	State random effects	All three years	−1.72 (0.36)***	Yes
6	Ecological	Percent change in state prevalence of underweight (1992–1998)	Percent change in per capita income (1998–1992)	None	1992, 1998	−2.40 (0.42)	No
7	Ecological	Percent change in state prevalence of underweight (1998–2005)	Percent change in per capita income (2005–1998)	None	1998, 2005	−56.40 (20.40)*	Yes
8	Ecological	Percent change in state prevalence of underweight (1992–2005)	Percent change in per capita income (2005–1992)	None	1992, 2005	−0.60 (4.20)	No
9	Multilevel	Individual probability of being underweight	Level of per capita income	None	Only 1992	0.83 (0.63, 1.11)	No
10	Multilevel	Individual probability of being underweight	Level of per capita income	All	Only 1992	0.99 (0.80, 1.22)	No
11	Multilevel	Individual probability of being underweight	Level of per capita income	None	Only 1998	0.84 (0.73, 0.97)	Yes
12	Multilevel	Individual probability of being underweight	Level of per capita income	All	Only 1998	0.99 (0.88, 1.11)	No
13	Multilevel	Individual probability of being underweight	Level of per capita income	None	Only 2005	0.94 (0.88, 1.01)	No
14	Multilevel	Individual probability of being underweight	Level of per capita income	All	Only 2005	1.01 (0.95, 1.07)	No
15	Multilevel	Individual probability of being underweight	Percent change in per capita income (2005–1992)	Level of per capita income in 1992	All three years	0.93 (0.76, 1.14)	No
16	Multilevel	Individual probability of being underweight	Percent change in per capita income (2005–1992)	None	All three years	0.94 (0.76, 1.16)	No
17	Multilevel	Individual probability of being underweight	Economic growth (change in per capita income)	All (no survey year)	All three years	0.94 (0.93, 0.96)	Yes
18	Multilevel	Individual probability of being underweight	Economic growth (change in per capita income)	All (plus survey year)	All three years	1.02 (0.99, 1.05)	No

a Level and change in per capita income measured in 5,000 INR. Percent change in per capita income measured in units of 60 percentage units.

b None  =  Includes state random effects. All  =  Age, gender, birth order, maternal age, maternal education, paternal education, religion, caste, urban residence, marital status, household wealth.

c Beta(s.e.) or OR (95% CI).

*p<0.05,

**p<0.01,

***p<0.001.

Economic growth was not associated with stunting, severe stunting, wasting, and severe wasting even when we used data only from 1998 and 2005 (which allowed us to include data from the five states that did not contain height data in 1992) (Table 5) as well as in models that accounted for sampling weights (Table S6).

**Table 5:** Odds ratios (95% confidence intervals) for associations between economic growth (in 5,000 INR over a 6.5-year period) and indicators of undernutrition from multilevel logistic models (using data from 24 states in 1998–99 and 2004–05).

	Stunting		Wasting	
	Any	Severe	Any	Severe
Year adjusted*	1.10 (1.05, 1.16)	1.14 (1.07, 1.21)	0.95 (0.91, 1.00)	1.00 (0.94, 1.07)
Year, age and sex adjusted*	1.12 (1.05, 1.18)	1.16 (1.08, 1.24)	0.96 (0.91, 1.01)	1.01 (0.94, 1.08)
Fully adjusted**	1.09 (1.04, 1.15)	1.11 (1.05, 1.18)	0.98 (0.93, 1.03)	1.03 (0.97, 1.09)

All estimates conditional on random effects.

*Additionally adjusted for state as *random* effect.

**Adjusted for state (as *random* effect), survey year, age, sex, birth order, maternal age, marital status, maternal education, paternal education, household wealth, caste, religion, and urban/rural residence.

The associations between undernutrition and the social factors included as covariates were in the expected direction (Table 6). Compared to children from households in the highest wealth quintile, children in the lowest quintile had an OR of 2.44 (95% CI 2.25, 2.65) of being underweight, an OR of 1.97 (95% CI 1.81, 2.13) of being stunted, and an OR of 1.64 (95% CI 1.48, 1.82) of being wasted. Similarly, we observed a gradient in the risk of being underweight across levels of maternal and paternal education. Compared to children of mothers with more than a college education, children of mothers who did not attend school had twice the odds of being underweight (OR = 2.04, 95% CI 1.82, 2.29), stunted (OR = 1.97, 95% CI 1.77, 2.20), and about 20% greater odds of being wasted (OR = 1.19, 95% CI 1.04, 1.38). The graded increase in the odds of being undernourished at lower levels of maternal education was clear for underweight and stunting but not for wasting. Similar patterns were observed with paternal education for all three undernutrition indicators.

**Table 6:** Odds ratios (95% confidence intervals) for associations between sociodemographic factors and indicators of undernutrition from multilevel logistic models.

Characteristic	Category	Underweight		Stunting		Wasting	
		Any	Severe	Any	Severe	Any	Severe
**Survey year**	1992	1.00	1.00	1.00	1.00	1.00	1.00
	1998	0.86 (0.80, 0.92)	0.82 (0.75, 0.90)	1.00 (0.93, 1.08)	0.96 (0.88, 1.04)	0.82 (0.74, 0.89)	0.76 (0.66, 0.87)
	2005	0.68 (0.61, 0.75)	0.56 (0.49, 0.64)	0.70 (0.64, 0.77)	0.56 (0.49, 0.63)	0.99 (0.87, 1.12)	0.85 (0.70, 1.02)
**Age in months**		1.25 (1.15, 1.37)	1.18 (1.06, 1.32)	1.57 (1.44, 1.73)	1.37 (1.24, 1.52)	1.11 (0.99, 1.23)	0.84 (0.69, 1.03)
**Gender**	Male	1.00	1.00	1.00	1.00	1.00	1.00
	Female	0.86 (0.83, 0.88)	0.90 (0.87, 0.94)	0.87 (0.85, 0.90)	0.84 (0.81, 0.87)	0.87 (0.84, 0.91)	0.83 (0.77, 0.88)
**Birth order**	First	1.00	1.00	1.00	1.00	1.00	1.00
	Second	1.03 (0.98, 1.07)	1.03 (0.98, 1.10)	1.01 (0.97, 1.06)	1.03 (0.97, 1.08)	1.13 (1.07, 1.20)	1.16 (1.06, 1.28)
	Third	1.04 (0.99, 1.10)	1.05 (0.98, 1.13)	1.00 (0.95, 1.05)	1.00 (0.93, 1.06)	1.16 (1.09, 1.25)	1.21 (1.08, 1.35)
	Fourth	1.07 (1.00, 1.14)	1.11 (1.02, 1.21)	0.95 (0.89, 1.01)	0.98 (0.91, 1.06)	1.25 (1.15, 1.36)	1.26 (1.10, 1.44)
	Fifth and greater	1.12 (1.05, 1.2)	1.30 (1.20, 1.42)	0.97 (0.90, 1.04)	1.06 (0.98, 1.15)	1.38 (1.26, 1.50)	1.32 (1.15, 1.51)
**Maternal age**	13 to 16 years	1.14 (0.98, 1.34)	1.06 (0.88, 1.28)	1.07 (0.90, 1.27)	0.93 (0.76, 1.13)	1.10 (0.90, 1.35)	1.13 (0.83, 1.54)
	17 to 19 years	1.00	1.00	1.00	1.00	1.00	1.00
	20 to 24 years	0.98 (0.93, 1.05)	0.95 (0.88, 1.03)	1.13 (1.06, 1.21)	1.12 (1.04, 1.21)	0.91 (0.84, 0.98)	0.85 (0.75, 0.97)
	25 to 29 years	1.00 (0.93, 1.07)	0.91 (0.83, 0.99)	1.17 (1.09, 1.25)	1.19 (1.09, 1.29)	0.82 (0.75, 0.89)	0.77 (0.67, 0.89)
	30 and more	1.05 (0.97, 1.13)	0.94 (0.86, 1.04)	1.28 (1.18, 1.39)	1.22 (1.11, 1.34)	0.81 (0.73, 0.89)	0.76 (0.65, 0.90)
**Maternal education**	No schooling	2.04 (1.82, 2.29)	1.99 (1.66, 2.39)	1.97 (1.77, 2.20)	2.11 (1.81, 2.46)	1.19 (1.04, 1.38)	1.04 (0.82, 1.32)
	Primary	1.87 (1.67, 2.11)	1.67 (1.39, 2.01)	1.87 (1.68, 2.08)	1.86 (1.60, 2.18)	1.14 (0.98, 1.31)	0.96 (0.76, 1.22)
	Secondary	1.55 (1.39, 1.73)	1.45 (1.22, 1.73)	1.56 (1.41, 1.72)	1.50 (1.30, 1.74)	1.06 (0.93, 1.21)	0.93 (0.75, 1.16)
	College	1.23 (1.09, 1.40)	1.13 (0.92, 1.39)	1.24 (1.10, 1.39)	1.13 (0.95, 1.34)	0.97 (0.83, 1.13)	0.99 (0.77, 1.27)
	>College	1.00	1.00	1.00	1.00	1.00	1.00
**Paternal education**	No schooling	1.37 (1.26, 1.48)	1.35 (1.21, 1.51)	1.36 (1.25, 1.47)	1.32 (1.19, 1.46)	1.22 (1.10, 1.35)	1.33 (1.12, 1.57)
	Primary	1.32 (1.22, 1.43)	1.22 (1.09, 1.37)	1.28 (1.18, 1.39)	1.27 (1.14, 1.40)	1.13 (1.02, 1.25)	1.13 (0.95, 1.34)
	Secondary	1.19 (1.11, 1.28)	1.11 (1.00, 1.23)	1.19 (1.11, 1.28)	1.14 (1.04, 1.25)	1.13 (1.03, 1.24)	1.08 (0.93, 1.26)
	College	1.07 (0.98, 1.16)	1.04 (0.92, 1.18)	1.11 (1.03, 1.20)	1.05 (0.95, 1.17)	1.06 (0.95, 1.18)	1.05 (0.87, 1.25)
	>College	1.00	1.00	1.00	1.00	1.00	1.00
**Marital status**	Married	1.00	1.00	1.00	1.00	1.00	1.00
	No longer married	1.05 (0.91, 1.22)	1.13 (0.94, 1.35)	1.10 (0.95, 1.27)	1.06 (0.89, 1.26)	1.10 (0.91, 1.32)	0.99 (0.73, 1.33)
**Household wealth**	Highest quintile	1.00	1.00	1.00	1.00	1.00	1.00
	Second quintile	1.51 (1.42, 1.60)	1.47 (1.34, 1.62)	1.41 (1.33, 1.50)	1.52 (1.40, 1.64)	1.18 (1.09, 1.28)	1.15 (1.00, 1.32)
	Third quintile	1.94 (1.81, 2.08)	1.95 (1.76, 2.15)	1.74 (1.63, 1.86)	2.01 (1.85, 2.20)	1.33 (1.22, 1.46)	1.34 (1.15, 1.55)
	Fourth quintile	2.25 (2.08, 2.42)	2.23 (2.01, 2.48)	1.89 (1.75, 2.03)	2.17 (1.97, 2.38)	1.48 (1.34, 1.63)	1.47 (1.25, 1.72)
	Lowest quintile	2.44 (2.25, 2.65)	2.50 (2.24, 2.80)	1.97 (1.81, 2.13)	2.37 (2.15, 2.62)	1.64 (1.48, 1.82)	1.55 (1.31, 1.84)
**Caste**	General	1.00	1.00	1.00	1.00	1.00	1.00
	Scheduled caste	1.34 (1.27, 1.42)	1.26 (1.17, 1.37)	1.28 (1.21, 1.35)	1.34 (1.25, 1.43)	1.13 (1.05, 1.21)	1.15 (1.02, 1.30)
	Scheduled tribe	1.28 (1.19, 1.38)	1.30 (1.19, 1.42)	1.18 (1.10, 1.27)	1.24 (1.14, 1.35)	1.25 (1.15, 1.37)	1.23 (1.07, 1.42)
	No caste	1.18 (1.12, 1.24)	1.11 (1.03, 1.19)	1.13 (1.07, 1.18)	1.17 (1.10, 1.24)	1.04 (0.97, 1.11)	1.03 (0.93, 1.15)
**Religion**	Hindu	1.00	1.00	1.00	1.00	1.00	1.00
	Muslim	1.04 (0.99, 1.10)	1.05 (0.98, 1.12)	1.07 (1.01, 1.13)	1.12 (1.05, 1.19)	1.00 (0.93, 1.07)	1.00 (0.89, 1.11)
	Christian	0.74 (0.67, 0.82)	0.70 (0.60, 0.82)	0.86 (0.78, 0.94)	0.90 (0.79, 1.02)	0.82 (0.72, 0.94)	0.90 (0.73, 1.11)
	Sikh	0.85 (0.73, 0.98)	0.87 (0.71, 1.06)	0.78 (0.68, 0.89)	0.70 (0.59, 0.84)	0.94 (0.77, 1.13)	0.76 (0.54, 1.07)
	Other	0.88 (0.77, 1.00)	0.99 (0.84, 1.18)	1.07 (0.95, 1.21)	1.00 (0.86, 1.17)	1.01 (0.86, 1.18)	0.87 (0.67, 1.12)
**Residence**	Urban	1.00	1.00	1.00	1.00	1.00	1.00
	Rural	0.96 (0.91, 1.01)	0.95 (0.89, 1.01)	0.96 (0.92, 1.01)	0.95 (0.89, 1.01)	1.00 (0.94, 1.06)	0.98 (0.88, 1.08)

All estimates conditional on random effects.

## Discussion

We found no consistent association between the risk of child undernutrition and state economic growth in India. A unique strength of our study was linking state economic growth to *individual* risk of undernutrition at the child level, and doing so with three repeated cross-sections of multilevel data. While we are not aware of any study examining this question in India or elsewhere, our findings are similar to those observed, albeit using cross-sectional multilevel data, on nutritional status of adult women in India, wherein no association was observed between state economic growth and risk of being underweight [35]. In one global ecologic study that used data from 63 countries over 26 years to examine a similar research question [3], it was shown that economic growth at the national level was inversely associated with risk of child undernutrition. The study also concluded that economic growth was responsible for about half the reduction in child undernutrition in that time period and that approximately half of this effect of economic growth was through increased food availability and the rest due to improvements in women's education, quality of health environment, and women's status. However, this is not directly comparable to our study primarily because it was an ecological study that did not account for individual-level factors. Among the eight ecological models we fit, we found support for the inverse association between economic growth (or per capita income in some cases) in only three models. It is important to be cautious while interpreting results from ecological studies in which both the outcome and exposure are measured at aggregate level as there is an assumption that the risk of undernutrition is the same for every child within a state/country. Such analyses are unable to measure the inherently *multilevel* association between economic growth, whether at the country or state level, and individual risk of undernutrition. Our multilevel findings, which are contrary to those reported in the between-country study, underscore the importance of avoiding generalizations from the country-level scale to the state-level, as well as the shortcomings of ecological analyses in examining a multilevel relationship.

Multilevel models correctly recognize that the likelihood of undernutrition can vary within a state between children, and also can vary between states. Critically, and very relevant to this study, multilevel models allow us to partition the variance in the probability of being undernourished into the part that is attributable to state-level factors and the part attributable to individual-level factors. They also enable us to quantify the association between a *state-level* exposure such as economic growth and an *individual-level* outcome such as the probability of being undernourished, having accounted for household, parental, and individual covariates. In only 2 of 10 multilevel models, we observed a support for an inverse association between per capita income (1998) or economic growth (all years) and undernutrition.

It must be noted that the multilevel model using data from three surveys that supported an inverse association does not account for survey-period differences. It is possible that the survey-period effects may capture changes unique to those years in programs that benefited from economic growth. Under the scenario where *all* consequential programs that could lead to reduction in child undernutrition were a result of *only* economic growth, our analysis suggests that a 5,000 INR increase in growth over 6.5 years is associated with a 4% to 7% reduced probability of undernutrition among children. However, this scenario is highly unlikely since a substantial number of programs are financed through the central government and/or by international agencies. In the context of our study, one example of unique changes in the 1998–99 survey period could be the Pulse Polio Campaign, an annual nationwide campaign to vaccinate all children under 5 years of age with Oral Polio Vaccine, which was initiated in 1995–96 [36]. This campaign was accompanied by substantial media coverage [37], with celebrities endorsing [38] visits to the local health centers and evaluations of the campaign including home visits by health professionals in certain areas. These annual campaigns impacted the health-seeking behaviors of caregivers of young children across the country and might have influenced the uptake of nutrition services, which would in turn impact the nutritional status of the children. Importantly, the funding for the Pulse Polio Campaign came from numerous international agencies such as the World Bank and UNICEF [39], in addition to the *central* government, and thus was not a result of state economic growth. Another example is the World Bank and UNICEF–funded Child Survival and Safe Motherhood (CSSM) Program, which was implemented from 1992–93 to 1997–98 and later merged into the Reproductive and Child Health (RCH) program in 1997–98 (http://mohfw.nic.in/dofw%20website/Child%20healthrti.pdf). The RCH differed from CSSM mainly in the approach towards delivery of services, and its implementation was widely publicized through mass media. Therefore, treating data from 1992 as equivalent to data from 1998 (or 2005) does not allow us to disentangle the effects of economic growth from the effect of other factors unique to any of the survey years that could be associated with reductions in child undernutrition. Further, it is incorrect to assume that data from 1992 are the same as data from 1998 (or 2005). Therefore, results from analyses excluding survey-period effects are inappropriate. At the same time, there might be concern that we may be explaining away the effects of economic growth by “over”-controlling, and for this reason we did not account for any changes driven by *state-level* economic growth, which is the focus of our analysis. One possible interpretation is that our results present a range for the possible effect sizes, from a 7% reduction in risk of child undernutrition over 7 years to no effect, suggesting that the association between economic growth and underweight is far from being a clear inverse association.

We posit the following as possible explanations for the lack of association between economic growth and undernutrition among children in India observed in our study. First, the pathways linking societal economic growth with individual well-being include that it increases the incomes of individuals and also a society's investment in public services, such as provision of clean drinking water and preventive health care, all of which are known to prevent undernutrition [5]. It is indeed possible that economic growth led to increased incomes and greater household wealth, which led to a reduced risk of undernutrition. The strong inverse association between household wealth and the risk of undernutrition during childhood appears to support this view. At the same time, there was no association between economic growth and childhood undernutrition even in models *without* household wealth or parental education, suggesting that this explanation is not supported by the data. On the other hand, a second explanation is that economic growth in India may have benefited only the privileged sections of society such that it translates into higher incomes among the better-off but not among the disadvantaged. This could lead to the lack of an association between economic growth and undernutrition. A direct test of the association between growth and differential increase in household wealth during 1992–2006 is not possible in our data because our measure of household wealth captures relative position of households on an asset scale and has no absolute interpretation. However, the documented increase in income inequality among Indian states in the 1992–2005 period [40] suggests that this might explain, at least in part, our null findings. Notably, Haryana and Meghalaya exhibited the highest rates of growth yet experienced high levels of undernutrition. Further research is needed to understand the processes unique to those states that might explain this remarkable finding.

Thirdly, there is some evidence that economic development is not a necessary condition to alleviate undernutrition in a population, the argument being that direct investment in preventive programs could also lead to improved nutritional status even in the absence of economic growth [8],[9]. State-level growth is an upstream determinant influencing individuals through multiple pathways such as investment in preventive and social programs targeting the nutritional status of children, or investment in better agricultural practices, which increase productivity, decrease food insecurity, and enable transfers of food-in-kind, or investment in infrastructure such as roads and colleges, which increases nutrition-related knowledge of caregivers and access to a variety of foods. Thus our null findings could be reflecting ineffective transfer along any/all of these pathways and not just through increased household wealth due to economic growth. It might even be argued that the gains, albeit modest, made in reducing undernutrition over the years are primarily a result of programs that intervene to improve health in general, for instance preventive health care [13],[14], and nutritional status in particular, such as the Integrated Child Services Development Scheme (ICDS), a national program focused on prevention and treatment of childhood undernutrition [19]. While the extensive primary health care system in India, ICDS, and other programs have no doubt made important contributions, existing evidence shows that the direct investment in preventive programs has been less than adequate [17], which perhaps explains the poor progress in reduction of child undernutrition in India [16]. Indeed, the relatively small reductions in the risk of child undernutrition over a period of 14 years are suggestive of the evidence that economic growth—directly or indirectly—has not translated to reductions in child undernutrition. Fourth, it is possible that economic growth over a 13-year period might not be sufficient to overcome the impact of intergenerational factors, such as the long-term and short-term nutritional status of mothers, on the risk of undernutrition among children [41]. Maternal height and body mass index have been shown to be important predictors of undernutrition in offspring both in India [18],[42] and in a majority of the developing countries [43]. Finally, it is possible that economic growth might indeed increase incomes of families; however, families might not use the additional income towards improving the nutritional status of their children. While we are unable to rule this out, there is some evidence that social cash interventions among the poor have resulted in, on average, greater use of health services and improved nutritional outcomes among children [44], suggesting that disadvantaged families are likely to invest in their children if economic growth resulted in increased income.

The following caveats need to be considered while interpreting our study. While there are several ways to measure nutritional status among children such as evaluating symptoms, functional performance, or laboratory assays of biomarkers, we chose to employ anthropometric indicators as per the WHO recommendation for epidemiological studies of undernutrition. We did so because they are the international standard for such studies and also because other measures of undernutrition were unavailable in these data [45]. Another limitation of our study is that the assessment of economic well-being at the household level was done via household wealth or asset index. While this measure captures the relative position of households with one another in each survey year, it does not have an absolute interpretation due to the manner of its construction, preventing us from directly testing whether economic growth was associated with differential improvement in household wealth in the 1992–2006 period. At the same time, this measure is widely acknowledged as a valid method of measuring household wealth in a developing country setting [30]. Since we found that the association between economic growth and undernutrition was not significant even prior to accounting for the effect of household wealth (or any other demographic and socioeconomic covariates), we believe that a lack of income measure may not be an explanation for the null association. A third limitation is that our models measure economic growth between survey periods and therefore not annual growth. Although we are using measurements from three points, each about 6.5 years apart, we believe that this is a reasonable representation of the pattern of economic growth of states in India between 1992 and 2005. Further investigation of annual per capita state income data revealed that there were less than six instances when per capita state income decreased among the 25 states over these 16 years. Fourthly, our measure of a state's income, the per capita net state domestic product, is only one measure of economic growth. Such measures have been described as tending to overestimate economic growth [26]. We have no reason to believe that any such issue varies across states. Additionally, the estimates produced by it correlate with estimates from other independent economic surveys [26]. It is also the indicator that is used widely as a measure of economic development of a state, including by the Government of India while allocating central resources to various states [27]. Price of food also varies across states. For instance, in 1999–2000, the Tornqvist Index, an index computed using expenditure on food, beverages, tobacco, and fuel, ranged from 92.4 to 123.2 in rural areas of states and 88.6 to 109.6 in the urban areas, although this has been described as “modest” spatial variation [46]. We were unable to account for this state-wise variation in prices, and consequently purchasing power, which might have influenced our results. Our data also had substantial missing data. However, the greatest number of missing records for wasting and stunting arose because height was not measured in five states in 1992. Among the 13,251 records missing data on stunting in 1992, 7,576 (about 57% of the missing) were due to non-measurement at the state-level as opposed to refusing measurement. Similarly about 56% of missing for wasting in 1992 was due to non-measurement. The figures for 1998 are 59% and 56% for 2005. The decision to not measure height of all children in these five states was based on logistics and not per capita income. Therefore the non-measurement is not related to our exposure of study and can be characterized as lack of data and not missing data due to non-response. There were greater missing data among children of mothers with no schooling, and states with lower per capita income were more likely to have a high proportion of such mothers. While we are unable to rule out the chance that this pattern of missingness has biased our results towards the null, it should be noted that models using data only from 2005–06, when the proportion of uneducated mothers was the same among those missing and not missing data, did not show any association between per capita income and undernutrition. Also, we have used the only available Indian data with objective measures of child undernutrition at the national level, from repeated surveys covering a 14-year period when India experienced rapid economic growth. We therefore feel that the strengths of our study outweigh the weaknesses. Finally, it is also necessary to examine whether these findings are generalizable to, and reproducible in, other countries that experienced rapid economic growth in the last two decades.

In summary, India is not on track for achieving the target for Millennium Development Goal (MDG) 4 of reducing child mortality [47]. Given that undernutrition between 6 and 59 months of age contributes to about 25% to 50% of the mortality in that age group [48], reducing undernutrition is imperative to achieving MDG 4. Indeed, reducing hunger constitutes the first MDG. The null association between state economic growth and undernutrition among children observed suggests that the role of economic growth and, more broadly, growth-mediated strategy in achieving the MDGs needs to be reappraised. The findings suggest that economic growth has no automatic connection to reducing childhood undernutrition. Further, reductions in the prevalence of childhood undernutrition in India are likely to depend on direct investments in health and health-related programs.

## Supporting Information

Table S1 Distribution of the prevalence of underweight, stunting, and wasting in the 1992-93, 1998-99, and 2005-06 INFHS surveys.
(0.22 MB DOC)

Table S2 Year-wise distribution of covariates in the Indian National Family Health survey data sets among children missing and not missing underweight data (*** *p*<0.0001, ** *p*<0.01, * *p*<0.05).
(0.10 MB DOC)

Table S3 Year-wise distribution of covariates in the Indian National Family Health survey data sets among children missing and not missing stunting data (*** *p*<0.0001, ** *p*<0.01, * *p*<0.05).
(0.10 MB DOC)

Table S4 Distribution of covariates in the Indian National Family Health survey data sets, among children missing and not missing wasting data, by survey year (*** *p*<0.0001, ** *p*<0.01, * *p*<0.05).
(0.10 MB DOC)

Table S5 State-wise proportion (%) of missing data for each survey year and the correlation of per capita state income (PCSI) and proportion of missing data in each state.
(0.08 MB DOC)

Table S6 Odds ratios (95% confidence intervals) for associations between economic growth, sociodemographic factors, and indicators of undernutrition from logistic models that account for sampling weights.
(0.07 MB DOC)

## Abbreviations

CSSM: Child Survival and Safe Motherhood
DHS: Demographic and Health Surveys
ICDS: Integrated Child Services Development Scheme
INR: Indian Rupees
MDG: Millennium Development Goal
NFHS: National Family Health Survey
OR: odds ratio
PPS: proportional to population size
RCH: Reproductive and Child Health
