# Supplementary material for: Is Economic Growth Associated with Reduction in Child Undernutrition in India?
Source: PLoS Med. 2011 Mar 8;8(3):e1000424. doi: 10.1371/journal.pmed.1000424 (PMC3050933; doi:10.1371/journal.pmed.1000424)
Supplement: Table S1 — Distribution of the prevalence of underweight, stunting, and wasting in the 1992-93, 1998-99, and 2005-06 INFHS surveys. (0.22 MB DOC) [file pmed.1000424.s001.doc]

**Table S1** Distribution of the prevalence of underweight, stunting and wasting in the 1992-93, 1998-99, and 2005-06 INFHS surveys

|  | **Underweight** | | | | | | | | **Stunted** | | | | | | | | **Wasted** | | | | | | | |
| --- | --- | --- | --- | --- | --- | --- | --- | --- | --- | --- | --- | --- | --- | --- | --- | --- | --- | --- | --- | --- | --- | --- | --- | --- |
|  | **Total** | | **1992-93** | | **1998-99** | | **2005-06** | | **Total** | | **1992-93** | | **1998-99** | | **2005-06** | | **Total** | | **1992-93** | | **1998-99** | | **2005-06** | |
| **Characteristic** | **N** | **Frequency (%)** | **N** | **Frequency (%)** | **N** | **Frequency (%)** | **N** | **Frequency (%)** | **N** | **Frequency (%)** | **N** | **Frequency (%)** | **N** | **Frequency (%)** | **N** | **Frequency (%)** | **N** | **Frequency (%)** | **N** | **Frequency (%)** | **N** | **Frequency (%)** | **N** | **Frequency (%)** |
| Survey year |  |  |  |  |  |  |  |  |  |  |  |  |  |  |  |  |  |  |  |  |  |  |  | |
| 1992-93 | 28066 | 12654 (49.15) |  |  |  |  |  |  | 20565 | 10208 (52.44) |  |  |  |  |  |  | 20580 | 4366 (24.01) |  |  |  |  |  |  |
| 1998-99 | 26121 | 10588 (43.82) |  |  |  |  |  |  | 18786 | 9265 (52.09) |  |  |  |  |  |  | 18718 | 3415(20.08) |  |  |  |  |  |  |
| 2005-06 | 23139 | 8034 (40.21) |  |  |  |  |  |  | 17423 | 7178 (45.93) |  |  |  |  |  |  | 17423 | 3345 (22.01) |  |  |  |  |  |  |
| Age (in months) |  |  |  |  |  |  |  |  |  |  |  |  |  |  |  |  |  |  |  |  |  |  |  |  |
| 0 to 11 | 25827 | 8424 (36.06) | 9660 | 3568 (40.67) | 8973 | 2896 (34.73) | 7194 | 1960 (31.95) | 18869 | 5179 (28.92) | 6983 | 2145 (31.99) | 6476 | 1867 (30.27) | 5410 | 1167 (24.15) | 18473 | 4347 (26.32) | 6818 | 1592 (26.40) | 6245 | 1397 (23.97) | 5410 | 1358 (28.48) |
| 12 to 23 | 26303 | 11322 (47.68) | 9708 | 4686 (52.70) | 8682 | 3711 (46.52) | 7913 | 2925 (43.17) | 19366 | 10368 (57.25) | 7182 | 3962 (57.97) | 6190 | 3496 (60.00) | 5994 | 2910 (54.14) | 19569 | 4017 (23.66) | 7291 | 1692 (26.86) | 6284 | 1175 (21.16) | 5994 | 1150 (22.40) |
| 24 to 35 | 25196 | 11530 (50.04) | 8698 | 4400 (54.60) | 8466 | 3981 (50.77) | 8032 | 3149 (44.84) | 18539 | 11104 (64.50) | 6400 | 4101 (68.90) | 6120 | 3902 (67.60) | 6019 | 3101 (57.77) | 18679 | 2762 (16.23) | 6471 | 1082 (18.11) | 6189 | 843 (14.91) | 6019 | 837 (15.59) |
| Gender |  |  |  |  |  |  |  |  |  |  |  |  |  |  |  |  |  |  |  |  |  |  |  |  |
| Male | 39931 | 16643 (45.57) | 14265 | 6735 (51.48) | 13656 | 5656 (44.34) | 12010 | 4252 (40.51) | 27547 | 14111 (51.21) | 10407 | 5361 (54.10) | 9799 | 4909 (52.78) | 9021 | 3841 (46.95) | 29314 | 5971 (22.92) | 10470 | 1964 (22.03) | 9823 | 1589 (19.75) | 9021 | 1743 (22.16) |
| Female | 37395 | 14633 (43.47) | 13801 | 5919 (46.80) | 12465 | 4932 (43.25) | 11129 | 3782 (39.88) | 29227 | 12540 (48.95) | 10158 | 4847 (50.76) | 8987 | 4356 (51.35) | 8402 | 3337 (44.81) | 27407 | 5155 (21.28) | 10110 | 2402 (25.94) | 8895 | 1826 (20.37) | 8402 | 1602 (21.84) |
| Birth order |  |  |  |  |  |  |  |  |  |  |  |  |  |  |  |  |  |  |  |  |  |  |  |  |
| First | 23091 | 8100 (39.23) | 7928 | 3235 (45.53) | 7654 | 2675 (38.34) | 7509 | 2190 (33.92) | 16473 | 6874 (45.07) | 5748 | 2629 (49.50) | 5278 | 2312 (46.37) | 5447 | 1933 (39.91) | 16386 | 2800 (19.37) | 5728 | 1035 (20.79) | 5211 | 853 (18.27) | 5447 | 912 (18.98) |
| Second | 20624 | 7630 (40.79) | 7013 | 2968 (46.33) | 6918 | 2534 (39.42) | 6693 | 2128 (38.73) | 14807 | 6565 (47.66) | 5085 | 2377 (49.51) | 4868 | 2279 (49.88) | 4854 | 1909 (44.03) | 14777 | 2760 (21.22) | 5071 | 1049 (23.60) | 4852 | 824 (18.54) | 4854 | 887 (21.31) |
| Third | 13408 | 5625 (45.45) | 5052 | 2258 (48.08) | 4608 | 1957 (45.54) | 3748 | 1410 (42.22) | 9944 | 4809 (50.65) | 3670 | 1843 (51.27) | 3395 | 1728 (53.64) | 2879 | 1238 (46.92) | 9905 | 1967 (22.39) | 3657 | 773 (24.19) | 3369 | 603 (19.70) | 2879 | 591 (23.11) |
| Fourth | 8067 | 3714 (50.75) | 3133 | 1562 (54.37) | 2786 | 1293 (50.34) | 2148 | 859 (46.42) | 6125 | 3119 (54.10) | 2340 | 1234 (55.54) | 2083 | 1126 (56.90) | 1702 | 759 (49.64) | 6142 | 1311 (23.57) | 2352 | 557 (26.75) | 2088 | 426 (22.15) | 1702 | 328 (21.09) |
| Fifth and higher | 12136 | 6207 (55.20) | 4940 | 2631 (56.06) | 4155 | 2129 (55.22) | 3041 | 1447 (53.40) | 9425 | 5284 (58.32) | 3722 | 2125 (59.02) | 3162 | 1820 (59.80) | 2541 | 1339 (56.14) | 9511 | 2288 (26.42) | 3772 | 952 (26.91) | 3198 | 709 (24.22) | 2541 | 627 (27.83) |
| Maternal age |  |  |  |  |  |  |  |  |  |  |  |  |  |  |  |  |  |  |  |  |  |  |  |  |
| <17 | 786 | 406 (52.68) | 316 | 170 (54.08) | 332 | 178 (54.75) | 138 | 58 (45.77) | 481 | 245 (50.77) | 199 | 104 (50.21) | 187 | 96 (51.07) | 95 | 45 (51.22) | 473 | 120 (26.70) | 195 | 44 (22.36) | 183 | 49 (28.20) | 95 | 27 (31.26) |
| 17-19 | 6427 | 2888 (46.72) | 2538 | 1239 (50.91) | 2378 | 1054 (45.90) | 1511 | 595 (42.04) | 4402 | 2139 (49.75) | 1710 | 851 (50.41) | 1588 | 780 (50.70) | 1104 | 508 (47.89) | 4369 | 974 (23.87) | 1692 | 391 (24.88) | 1573 | 346 (22.63) | 1104 | 237 (24.02) |
| 20-24 | 28959 | 11511 (16.79) | 10504 | 4652 (47.76) | 9789 | 3880 (41.95) | 8666 | 2979 (38.00) | 20621 | 9624 (48.90) | 7514 | 3705 (51.76) | 6870 | 3352 (51.02) | 6237 | 2567 (44.19) | 20531 | 4046 (21.93) | 7495 | 1562 (23.72) | 6799 | 1255 (20.06) | 6237 | 1229 (21.77) |
| 25-29 | 24360 | 9390 (43.04) | 8509 | 3675 (47.83) | 8239 | 3176 (42.27) | 7612 | 2539 (38.87) | 18062 | 8167 (48.72) | 6291 | 3007 (50.55) | 6021 | 2892 (51.35) | 5750 | 2268 (44.63) | 18086 | 3336 (20.75) | 6307 | 1288 (23.17) | 6029 | 978 (17.94) | 5750 | 1070 (20.92) |
| >29 | 16794 | 7081 (49.01) | 6199 | 2918 (52.29) | 5383 | 2300 (48.17) | 5212 | 1863 (45.88) | 13208 | 6476 (54.43) | 4851 | 2541 (56.91) | 4120 | 2145 (56.16) | 4237 | 1790 (50.17) | 13262 | 2650 (23.45) | 4891 | 1081 (25.23) | 4134 | 787 (21.62) | 4237 | 782 (22.98) |
| Marital status |  |  |  |  |  |  |  |  |  |  |  |  |  |  |  |  |  |  |  |  |  |  |  |  |
| Married | 76418 | 30908 (44.53) | 27735 | 12509 (49.15) | 25806 | 10462 (43.79) | 22877 | 7937 (40.16) | 56102 | 26313 (50.05) | 20323 | 10079 (52.36) | 18551 | 9143 (52.05) | 17228 | 7091 (45.88) | 56040 | 10985 (22.14) | 20332 | 4317 (24.03) | 18480 | 3370 (20.11) | 17228 | 3298 (21.98) |
| Unmarried | 908 | 368 (46.75) | 331 | 145 (49.11) | 315 | 126 (45.93) | 262 | 97 (45.02) | 672 | 338 (56.80) | 242 | 129 (61.45) | 235 | 122 (56.12) | 195 | 87 (52.12) | 681 | 141 (21.28) | 248 | 49 (21.80) | 238 | 45 (17.14) | 195 | 47 (25.68) |
| Maternal education |  |  |  |  |  |  |  |  |  |  |  |  |  |  |  |  |  |  |  |  |  |  |  |  |
| None | 37995 | 19381 (53.43) | 16104 | 8643 (55.99) | 12923 | 6586 (53.29) | 8968 | 4152 (49.87) | 28182 | 15742 (57.55) | 11602 | 6597 (58.20) | 9570 | 5555 (60.07) | 7010 | 3590 (54.23) | 28259 | 6636 (25.17) | 11677 | 2872 (26.27) | 9572 | 2059 (22.77) | 7010 | 1705 (26.06) |
| 1-5 | 11089 | 4499 (43.54) | 3803 | 1618 (46.10) | 4097 | 1657 (43.56) | 3189 | 1224 (40.96) | 7525 | 3626 (49.68) | 2635 | 1303 (50.12) | 2667 | 1336 (52.36) | 2223 | 987 (46.60) | 7505 | 1416 (21.41) | 2623 | 527 (23.55) | 2659 | 457 (19.25) | 2223 | 432 (21.45) |
| 6-10 | 20206 | 5982 (32.99) | 6098 | 1953 (36.25) | 6699 | 1934 (31.35) | 7409 | 2095 (32.00) | 15062 | 5760 (40.99) | 4765 | 1862 (41.83) | 4802 | 1908 (42.38) | 5495 | 1990 (39.28) | 14975 | 2356 (18.09) | 4725 | 766 (19.48) | 4755 | 709 (16.52) | 5495 | 881 (18.30) |
| 11-12 | 3843 | 790 (23.78) | 978 | 239 (28.51) | 1185 | 235 (23.91) | 1680 | 316 (20.64) | 2893 | 837 (31.09) | 729 | 212 (31.91) | 877 | 271 (33.17) | 1287 | 354 (29.10) | 2878 | 379 (15.23) | 723 | 108 (18.31) | 868 | 110 (15.65) | 1287 | 161 (13.21) |
| >12 | 4193 | 624 (16.67) | 1083 | 201 (20.78) | 1217 | 176 (16.67) | 1893 | 247 (14.05) | 3112 | 686 (22.79) | 834 | 234 (30.35) | 870 | 195 (22.61) | 1408 | 257 (18.39) | 3104 | 339 (12.88) | 832 | 93 (12.78) | 864 | 80 (12.11) | 1408 | 166 (13.47) |
| Paternal education |  |  |  |  |  |  |  |  |  |  |  |  |  |  |  |  |  |  |  |  |  |  |  |  |
| None | 20869 | 10945 (54.70) | 8866 | 4872 (57.08) | 6859 | 3603 (54.95) | 5144 | 2470 (51.06) | 14956 | 8542 (58.66) | 6140 | 3528 (58.73) | 4936 | 2939 (61.36) | 3880 | 2075 (56.05) | 15036 | 3738 (26.40) | 6212 | 1583 (27.11) | 4944 | 1150 (24.66) | 3880 | 1005 (27.10) |
| 1-5 | 12074 | 5573 (49.63) | 4461 | 2217 (52.27) | 4323 | 1997 (49.66) | 3290 | 1359 (46.41) | 8311 | 4251 (54.03) | 3084 | 1648 (55.43) | 2908 | 1505 (54.60) | 2319 | 1098 (51.86) | 8297 | 1760 (23.86) | 3074 | 686 (24.24) | 2904 | 593 (22.48) | 2319 | 481 (24.83) |
| 6-10 | 29645 | 10934 (41.02) | 10181 | 4128 (45.58) | 10079 | 3727 (39.82) | 9385 | 3079 (37.79) | 22553 | 10120 (48.26) | 7920 | 3707 (50.31) | 7442 | 3517 (50.47) | 7191 | 2896 (44.41) | 22477 | 4078 (20.86) | 7885 | 1527 (22.82) | 7401 | 1236 (18.64) | 7191 | 1315 (20.94) |
| 11-12 | 6709 | 2016 (34.42) | 2061 | 726 (40.01) | 2314 | 688 (32.84) | 2334 | 602 (31.03) | 4991 | 1947 (41.81) | 1553 | 674 (44.40) | 1646 | 691 (44.67) | 1792 | 582 (37.18) | 4974 | 762 (17.67) | 1548 | 286 (21.43) | 1634 | 214 (14.56) | 1792 | 262 (17.27) |
| >12 | 8029 | 1808 (25.37) | 2497 | 711 (31.70) | 2546 | 573 (25.50) | 2986 | 524 (19.73) | 5963 | 1791 (32.89) | 1868 | 651 (38.33) | 1854 | 613 (35.33) | 2241 | 527 (26.56) | 5937 | 788 (15.32) | 1861 | 284 (18.47) | 1835 | 222 (14.02) | 2241 | 282 (13.88) |
| Wealth quintile |  |  |  |  |  |  |  |  |  |  |  |  |  |  |  |  |  |  |  |  |  |  |  |  |
| Highest quintile | 14743 | 3147 (23.04) | 4820 | 1273 (29.32) | 5050 | 1058 (22.14) | 4873 | 816 (17.59) | 11281 | 3466 (31.91) | 3835 | 1332 (36.07) | 3732 | 1241 (34.68) | 3714 | 893 (25.40) | 11219 | 1432 (14.24) | 3799 | 557 (17.52) | 3706 | 418 (12.35) | 3714 | 457 (13.11) |
| Second quintile | 17330 | 5782 (35.28) | 5750 | 2127 (39.33) | 6229 | 2156 (36.03) | 5351 | 1499 (30.42) | 13087 | 5610 (44.84) | 4501 | 2066 (47.99) | 4534 | 2058 (47.27) | 4052 | 1486 (39.44) | 13017 | 2105 (18.10) | 4463 | 775 (20.01) | 4502 | 662 (15.71) | 4052 | 668 (18.66) |
| Third quintile | 15974 | 6780 (45.03) | 5725 | 2702 (50.44) | 5493 | 2382 (45.67) | 4756 | 1696 (38.60) | 11561 | 5803 (51.62) | 3986 | 2078 (52.95) | 3899 | 2078 (54.48) | 3676 | 1647 (47.69) | 11544 | 2267 (21.71) | 3968 | 847 (23.66) | 3900 | 726 (20.66) | 3676 | 694 (20.82) |
| Fourth quintile | 14722 | 7362 (52.09) | 5561 | 2961 (55.70) | 4911 | 2460 (51.61) | 4250 | 1941 (48.52) | 10575 | 5746 (55.66) | 3917 | 2179 (56.50) | 3399 | 1955 (59.49) | 3259 | 1612 (51.62) | 10599 | 2450 (24.96) | 3961 | 986 (26.46) | 3379 | 703 (22.02) | 3259 | 761 (25.81) |
| Lowest quintile | 14557 | 8205 (57.40) | 6210 | 3591 (58.57) | 4438 | 2532 (58.44) | 3909 | 2082 (55.05) | 10270 | 6026 (60.06) | 4326 | 2553 (60.01) | 3222 | 1933 (61.94) | 2722 | 1540 (58.60) | 10342 | 2872 (28.29) | 4389 | 1201 (28.04) | 3231 | 906 (28.62) | 2722 | 765 (28.35) |
| Caste |  |  |  |  |  |  |  |  |  |  |  |  |  |  |  |  |  |  |  |  |  |  |  |  |
| Scheduled caste | 12588 | 5922 (49.26) | 3631 | 1888 (54.17) | 4826 | 2275 (48.68) | 4131 | 1759 (46.41) | 8965 | 4863 (56.22) | 2663 | 1498 (57.70) | 3331 | 1886 (58.29) | 2971 | 1479 (53.48) | 8966 | 1974 (23.75) | 2663 | 663 (27.15) | 3332 | 663 (20.36) | 2971 | 648 (24.33) |
| Scheduled tribe | 10848 | 4442 (54.48) | 3449 | 1546 (55.21) | 3842 | 1588 (55.70) | 3557 | 1308 (52.35) | 8362 | 3827 (53.48) | 2397 | 1151 (52.88) | 3057 | 1487 (57.74) | 2908 | 1189 (49.45) | 8338 | 1709 (28.86) | 2409 | 517 (29.54) | 3021 | 600 (28.67) | 2908 | 592 (28.36) |
| No caste | 1164 | 363 (39.11) |  |  | 149 | 68 (48.62) | 1015 | 295 (36.82) | 971 | 355 (44.08) |  |  | 136 | 76 (58.48) | 835 | 279 (38.79) | 970 | 152 (18.35) |  |  | 135 | 28 (20.67) | 835 | 124 (17.651) |
| General caste | 52726 | 20549 (42.21) | 20986 | 9220 (47.62) | 17304 | 6657 (40.70) | 14436 | 4672 (36.81) | 38476 | 17606 (48.36) | 15505 | 7559 (51.52) | 12262 | 5816 (49.53) | 10709 | 4231 (43.56) | 38447 | 7291 (21.02) | 15508 | 3186 (22.92) | 12230 | 2124 (18.83) | 10709 | 1981 (20.77) |
| Religion |  |  |  |  |  |  |  |  |  |  |  |  |  |  |  |  |  |  |  |  |  |  |  |  |
| Hindu | 56826 | 24194 (45.29) | 21321 | 9938 (49.58) | 19467 | 8371 (44.78) | 16038 | 5885 (41.03) | 39799 | 19288 (50.69) | 15094 | 7629 (52.69) | 13328 | 6848 (53.31) | 11377 | 4811 (46.27) | 39743 | 8214 (22.65) | 15121 | 3361 (24.53) | 13245 | 2577 (20.80) | 11377 | 2276 (22.36) |
| Muslim | 11214 | 4655 (44.82) | 3726 | 1807 (51.56) | 3710 | 1523 (43.86) | 3778 | 1325 (38.87) | 8506 | 4141 (51.01) | 2762 | 1496 (55.63) | 2839 | 1400 (51.22) | 2905 | 1245 (46.30) | 8549 | 1686 (21.30) | 2766 | 589 (23.36) | 2878 | 528 (19.10) | 2905 | 569 (21.16) |
| Christian | 5815 | 1358 (28.09) | 1714 | 430 (29.18) | 1815 | 397 (26.57) | 2286 | 531 (28.77) | 5279 | 1919 (35.29) | 1521 | 549 (34.16) | 1601 | 610 (34.95) | 2157 | 760 (36.96) | 5239 | 723 (15.71) | 1507 | 189 (13.90) | 1575 | 207 (14.56) | 2157 | 327 (18.97) |
| Sikh | 1862 | 538 (29.15) | 839 | 295 (36.74) | 574 | 147 (25.67) | 449 | 96 (21.41) | 1779 | 675 (37.64) | 815 | 332 (40.86) | 531 | 224 (42.10) | 433 | 119 (27.99) | 1762 | 235 (13.37) | 798 | 143 (18.53) | 531 | 39 (7.77) | 433 | 53 (11.50) |
| Other/missing data | 1609 | 531 (42.99) | 466 | 184 (45.22) | 555 | 150 (39.01) | 588 | 197 (44.33) | 1411 | 628 (49.55) | 373 | 202 (52.62) | 487 | 183 (38.51) | 551 | 243 (55.41) | 1428 | 268 (25.22) | 388 | 84 (27.31) | 489 | 64 (18.82) | 551 | 120 (28.41) |
| Type of residence |  |  |  |  |  |  |  |  |  |  |  |  |  |  |  |  |  |  |  |  |  |  |  |  |
| Urban | 23368 | 7413 (34.88) | 7750 | 2856 (40.15) | 7072 | 2235 (34.38) | 8546 | 2322 (29.79) | 17055 | 6859 (42.66) | 5966 | 2630 (46.00) | 4992 | 2063 (43.67) | 6097 | 5012 (48.23) | 16994 | 2748 (18.35) | 5936 | 1061 (21.34) | 4961 | 724 (15.99) | 6097 | 963 (17.43) |
| Rural | 53958 | 23863 (47.53) | 20316 | 9798 (51.86) | 19049 | 8353 (46.66) | 14593 | 5712 (43.55) | 39719 | 19792 (52.35) | 14599 | 7578 (54.35) | 13794 | 7202 (54.63) | 11326 | 2166 (38.36) | 39727 | 8378 (23.26) | 14644 | 3305 (24.80) | 13757 | 2691 (21.30) | 11326 | 2382 (23.41) |
| **Total** | **####** | **31276 (44.55)** | **####** | **12654 (49.15)** | **####** | **10588 (43.82)** | **####** | **8034 (40.21)** | **####** | **26651 (50.11)** | **####** | **10208 (52.44)** | **####** | **9265 (52.09)** | **####** | **7178 (45.93)** | **####** | **11126 (22.13)** | **####** | **4366 (24.01)** | **####** | **3415 (20.08)** | **####** | **3345 (22.01)** |
