# Supplementary material for: Is Economic Growth Associated with Reduction in Child Undernutrition in India?
Source: PLoS Med. 2011 Mar 8;8(3):e1000424. doi: 10.1371/journal.pmed.1000424 (PMC3050933; doi:10.1371/journal.pmed.1000424)
Supplement: Table S2 — Year-wise distribution of covariates in the Indian National Family Health survey data sets among children missing and not missing underweight data (*** p<0.0001, ** p<0.01, * p<0.05). (0.10 MB DOC) [file pmed.1000424.s002.doc]

**Table S2:** Year-wise distribution of covariates in the Indian National Family Health survey datasets among children missing and not missing underweight data

(*** = p <0.0001, **= p< 0.01 and * = p<0.05)

|  |  | **1992-93** | | **1998-99** | | **2005-06** | |
| --- | --- | --- | --- | --- | --- | --- | --- |
| **Characteristic** |  | **Non-missing** | **Missing** | **Non-missing** | **Missing** | **Non-missing** | **Missing** |
| Age (in months) | 0 to 11 | 34.33 | 38.5*** | 34.45 | 33.73** | 31.47 | 44.72*** |
|  | 12 to 23 | 35.02 | 29.99 | 33.57 | 31.04 | 34.41 | 27.71 |
|  | 24 to 35 | 30.65 | 31.51 | 31.98 | 35.23 | 34.12 | 27.57 |
| Gender | Male | 50.42 | 52.42* | 51.89 | 52.31 | 52.18 | 53.42 |
|  | Female | 49.58 | 47.59 | 48.11 | 47.69 | 47.82 | 46.58 |
| Birth order | First | 27.38 | 28.28 | 29.41 | 26.48*** | 30.92 | 31.6 |
|  | Second | 24.5 | 23.23 | 26.48 | 23.29 | 28.04 | 27.72 |
|  | Third | 17.97 | 17.26 | 18.03 | 16.72 | 16.3 | 15.43 |
|  | Fourth | 11.5 | 11.85 | 10.51 | 10.37 | 9.61 | 9.58 |
|  | Fifth and higher | 18.66 | 19.38 | 15.59 | 23.14 | 15.13 | 15.66 |
| Maternal age | <17 | 1.38 | 1.65 | 1.69 | 1.78*** | 0.82 | 0.98 |
|  | 17-19 | 10.49 | 11.01 | 10.94 | 10.82 | 8.47 | 10.04 |
|  | 20-24 | 38.35 | 37.49 | 39.33 | 35.82 | 40.5 | 38.48 |
|  | 25-29 | 28.68 | 28.02 | 29.97 | 28.32 | 30.76 | 29.89 |
|  | >29 | 21.11 | 21.83 | 18.08 | 23.26 | 19.45 | 20.61 |
| Marital status | Married | 99.05 | 98.99 | 98.92 | 98.58 | 99.08 | 99.08 |
|  | Unmarried | 0.95 | 1.01 | 1.08 | 1.42 | 0.92 | 0.92 |
| Maternal education | None | 62.71 | 67.58*** | 52.28 | 65.59*** | 47.7 | 47.03* |
|  | 1-5 | 12.59 | 11 | 15.64 | 11.35 | 13.88 | 12.73 |
|  | 6-10 | 18.52 | 15.81 | 23.75 | 15.72 | 27.77 | 27.98 |
|  | 11-12 | 3.13 | 2.83 | 4.35 | 3.7 | 5.39 | 5.24 |
|  | >12 | 3.05 | 2.79 | 3.99 | 3.65 | 5.26 | 7.02 |
| Paternal education | None | 34.84 | 38.24** | 28.33 | 35.25*** | 27.11 | 33.05*** |
|  | 1-5 | 16.43 | 15.63 | 17.07 | 14.46 | 15.02 | 13.06 |
|  | 6-10 | 33.38 | 31.78 | 36.28 | 32.64 | 38.56 | 34.23 |
|  | 11-12 | 7.2 | 7.44 | 8.92 | 9.18 | 8.98 | 8.2 |
|  | >12 | 8.15 | 6.91 | 9.41 | 8.47 | 10.33 | 1.46 |
| Wealth quintile | Highest quintile | 13.31 | 12.42** | 15.89 | 13.24*** | 14.34 | 18.09** |
|  | Second quintile | 17.08 | 14.73 | 20.85 | 16.23 | 19.02 | 18.49 |
|  | Third quintile | 19.45 | 18.58 | 21.12 | 19.28 | 20.15 | 17.35 |
|  | Fourth quintile | 22.92 | 25.14 | 21.78 | 24.9 | 22.56 | 21.45 |
|  | Lowest quintile | 27.24 | 29.13 | 20.36 | 26.36 | 23.94 | 24.62 |
| Caste | Scheduled caste | 12.99 | 13.22* | 19.89 | 18.67*** | 20.74 | 20.71 |
|  | Scheduled tribe | 9 | 10.74 | 9.8 | 6.91 | 9.06 | 10.47 |
|  | No caste | NA | NA | 0.78 | 3.01 | 3.24 | 2.91 |
|  | General caste | 78.01 | 76.04 | 69.52 | 71.41 | 66.95 | 65.91 |
| Religion | Hindu | 79.43 | 78.08* | 79.52 | 76.11*** | 78.6 | 75.57** |
|  | Muslim | 15.18 | 16.41 | 15.07 | 20.83 | 16.57 | 19.78 |
|  | Christian | 2.04 | 2.16 | 2.53 | 1.38 | 1.99 | 2.34 |
|  | Sikh | 1.91 | 1.41 | 1.5 | 0.89 | 1.38 | 0.76 |
|  | Other/missing data | 1.45 | 1.94 | 1.38 | 0.79 | 1.46 | 1.55 |
| Type of residence | Urban | 23.12 | 23.53 | 23.17 | 19.3** | 24.26 | 34.69*** |
|  | Rural | 76.88 | 76.47 | 76.83 | 80.7 | 75.74 | 65.31 |
