# Supplementary material for: Is Economic Growth Associated with Reduction in Child Undernutrition in India?
Source: PLoS Med. 2011 Mar 8;8(3):e1000424. doi: 10.1371/journal.pmed.1000424 (PMC3050933; doi:10.1371/journal.pmed.1000424)
Supplement: Table S3 — Year-wise distribution of covariates in the Indian National Family Health survey data sets among children missing and not missing stunting data (*** p<0.0001, ** p<0.01, * p<0.05). (0.10 MB DOC) [file pmed.1000424.s003.doc]

**Table S3:** Year-wise distribution of covariates in the Indian National Family Health survey datasets among children missing and not missing stunting data

(*** = p <0.0001, **= p< 0.01 and * = p<0.05)

|  |  | **1992-93** | | **1998-99** | | **2005-06** | |
| --- | --- | --- | --- | --- | --- | --- | --- |
| **Characteristic** |  | **Non-missing** | **Missing** | **Non-missing** | **Missing** | **Non-missing** | **Missing** |
| Age (in months) | 0 to 11 | 34.09 | 38.08*** | 34.80 | 32.53** | 31.46 | 43.01*** |
|  | 12 to 23 | 35.48 | 30.79 | 33.17 | 31.77 | 34.75 | 28.05 |
|  | 24 to 35 | 30.43 | 31.13 | 32.04 | 35.70 | 33.78 | 28.94 |
| Gender | Male | 50.28 | 53.77** | 51.87 | 53.45 | 52.35 | 53.39 |
|  | Female | 49.72 | 46.23 | 48.13 | 46.55 | 47.65 | 46. 61 |
| Birth order | First | 26.37 | 26.65** | 27.69 | 24.62*** | 29.57 | 30.41 |
|  | Second | 23.91 | 21.46 | 25.38 | 23.00 | 26.61 | 26.16 |
|  | Third | 17.83 | 17.44 | 18.79 | 16.76 | 16.57 | 15.79 |
|  | Fourth | 11.9 | 12.27 | 10.96 | 10.98 | 10.13 | 10.03 |
|  | Fifth and higher | 20 | 22.19 | 17.18 | 24.67 | 17.11 | 17.61 |
| Maternal age | <17 | 1.14 | 1.54 | 1.33 | 1.48*** | 0.70 | 0.81 |
|  | 17-19 | 9.62 | 10.19 | 10.16 | 10.75 | 8.24 | 9.67 |
|  | 20-24 | 37.66 | 35.20 | 38.38 | 34.40 | 39.11 | 37.38 |
|  | 25-29 | 28.68 | 28.97 | 30.67 | 29.38 | 31.11 | 30.01 |
|  | >29 | 22.89 | 24.10 | 19.47 | 24.00 | 20.83 | 22.14 |
| Marital status | Married | 99.11 | 99.02 | 98.98 | 98.70 | 99.22 | 99.25 |
|  | Unmarried | 0.89 | 0.98 | 1.02 | 1.30 | 0.78 | 0.75 |
| Maternal education | None | 63.19 | 71.53*** | 54.61 | 67.36*** | 50.09 | 49.98 |
|  | 1-5 | 11.28 | 8.85 | 13.08 | 10.23 | 11.93 | 10.73 |
|  | 6-10 | 19.31 | 14.31 | 23.45 | 15.26 | 26.86 | 27.47 |
|  | 11-12 | 3.1 | 2.35 | 4.71 | 3.68 | 5.71 | 5.12 |
|  | >12 | 3.13 | 2.96 | 4.16 | 3.46 | 5.41 | 6.70 |
| Paternal education | None | 33.24 | 39.27*** | 27.86 | 35.39*** | 26.96 | 33.00*** |
|  | 1-5 | 15.08 | 14.15 | 14.92 | 13.59 | 13.29 | 12.14 |
|  | 6-10 | 35.82 | 32.95 | 38.21 | 33.76 | 39.52 | 35.09 |
|  | 11-12 | 7.55 | 6.87 | 9.13 | 9.12 | 9.45 | 8.76 |
|  | >12 | 8.32 | 6.76 | 9.87 | 8.14 | 10.79 | 11.02 |
| Wealth quintile | Highest quintile | 13.95 | 11.71*** | 17.10 | 12.31*** | 15.75 | 18.64** |
|  | Second quintile | 17.85 | 14.39 | 21.55 | 16.62 | 19.44 | 19.59 |
|  | Third quintile | 18.31 | 17.50 | 20.45 | 18.95 | 19.75 | 16.16 |
|  | Fourth quintile | 22.55 | 25.38 | 20.59 | 24.61 | 22.42 | 20.70 |
|  | Lowest quintile | 27.34 | 31.03 | 20.31 | 27.51 | 22.65 | 24.91 |
| Caste | Scheduled caste | 13.16 | 13.38** | 19.18 | 19.34*** | 20.48 | 19.93** |
|  | Scheduled tribe | 7.97 | 9.92 | 9.56 | 6.74 | 7.86 | 10.12 |
|  | No caste | NA | NA | 1.06 | 2.95 | 2.59 | 2.71 |
|  | General caste | 78.87 | 76.70 | 70.21 | 70.98 | 69.08 | 67.24 |
| Religion | Hindu | 77.57 | 76.85** | 78.27 | 73.98*** | 77.78 | 73.53*** |
|  | Muslim | 15.86 | 17.42 | 15.72 | 22.25 | 16.68 | 21.40 |
|  | Christian | 2.11 | 1.98 | 2.21 | 1.74 | 1.88 | 2.18 |
|  | Sikh | 2.72 | 1.6 | 2.17 | 1.11 | 1.83 | 0.95 |
|  | Other/missing data | 1.75 | 2.16 | 1.64 | 0.92 | 1.83 | 1.93 |
| Type of residence | Urban | 22.88 | 21.48 | 23.14 | 17.75** | 23.33 | 35.24*** |
|  | Rural | 77.13 | 78.52 | 76.86 | 82.25 | 76.67 | 64.76 |
