# Supplementary material for: Is Economic Growth Associated with Reduction in Child Undernutrition in India?
Source: PLoS Med. 2011 Mar 8;8(3):e1000424. doi: 10.1371/journal.pmed.1000424 (PMC3050933; doi:10.1371/journal.pmed.1000424)
Supplement: Table S4 — Distribution of covariates in the Indian National Family Health survey data sets, among children missing and not missing wasting data, by survey year (*** p<0.0001, ** p<0.01, * p<0.05). (0.10 MB DOC) [file pmed.1000424.s004.doc]

**Table S4:** Distribution of covariates in the Indian National Family Health survey datasets, among children missing and not missing wasting data, by survey year (*** = p <0.0001, **= p< 0.01 and * = p<0.05)

|  |  | **1992-93** | | **1998-99** | | **2005-06** | |
| --- | --- | --- | --- | --- | --- | --- | --- |
| **Characteristic** |  | **Non-missing** | **Missing** | **Non-missing** | **Missing** | **Non-missing** | **Missing** |
| Age (in months) | 0 to 11 | 33.21 | 41.88*** | 33.63 | 37.02*** | 31.46 | 43.01*** |
|  | 12 to 23 | 35.95 | 28.69 | 33.85 | 29.17 | 34.75 | 28.05 |
|  | 24 to 35 | 30.83 | 29.43 | 32.51 | 33.82 | 33.78 | 28.94 |
| Gender | Male | 50.62 | 52.37 | 52.12 | 52.47 | 52.35 | 53.39 |
|  | Female | 49.38 | 47.63 | 47.88 | 47.53 | 47.65 | 46. 61 |
| Birth order | First | 26.32 | 26.89 | 27.38 | 25.84*** | 29.57 | 30.41 |
|  | Second | 23.73 | 22.14 | 25.37 | 23.06 | 26.61 | 26.16 |
|  | Third | 17.76 | 17.70 | 18.69 | 17.19 | 16.57 | 15.79 |
|  | Fourth | 11.89 | 12.32 | 11.12 | 10.35 | 10.13 | 10.03 |
|  | Fifth and higher | 20.30 | 20.95 | 17.45 | 23.57 | 17.11 | 17.61 |
| Maternal age | <17 | 1.09 | 1.74** | 1.30 | 1.58** | 0.70 | 0.81 |
|  | 17-19 | 9.51 | 10.69 | 10.09 | 11.04 | 8.24 | 9.67 |
|  | 20-24 | 37.53 | 35.74 | 38.13 | 35.40 | 39.11 | 37.38 |
|  | 25-29 | 28.68 | 29.01 | 30.90 | 28.51 | 31.11 | 30.01 |
|  | >29 | 23.20 | 22.83 | 19.59 | 23.47 | 20.83 | 22.14 |
| Marital status | Married | 99.09 | 99.10 | 98.94 | 98.90 | 99.22 | 99.25 |
|  | Unmarried | 0.91 | 0.90 | 1.06 | 1.10 | 0.78 | 0.75 |
| Maternal education | None | 63.54 | 70.21*** | 54.81 | 66.45*** | 50.09 | 49.98 |
|  | 1-5 | 11.24 | 9.00 | 13.07 | 10.31 | 11.93 | 10.73 |
|  | 6-10 | 19.09 | 15.14 | 23.30 | 15.92 | 26.86 | 27.47 |
|  | 11-12 | 3.06 | 2.53 | 4.70 | 3.77 | 5.71 | 5.12 |
|  | >12 | 3.09 | 3.13 | 4.14 | 3.57 | 5.41 | 6.70 |
| Paternal education | None | 33.59 | 37.92** | 27.99 | 34.78*** | 26.96 | 33.00*** |
|  | 1-5 | 15.04 | 14.28 | 14.98 | 13.41 | 13.29 | 12.14 |
|  | 6-10 | 35.65 | 33.60 | 38.12 | 34.17 | 39.52 | 35.09 |
|  | 11-12 | 7.48 | 7.15 | 9.10 | 9.26 | 9.45 | 8.76 |
|  | >12 | 8.24 | 7.06 | 9.81 | 8.39 | 10.79 | 11.02 |
| Wealth quintile | Highest quintile | 13.74 | 12.55** | 17.02 | 12.67*** | 15.75 | 18.64** |
|  | Second quintile | 17.76 | 14.73 | 21.57 | 16.58 | 19.44 | 19.59 |
|  | Third quintile | 18.23 | 17.81 | 20.51 | 18.74 | 19.75 | 16.16 |
|  | Fourth quintile | 22.64 | 25.04 | 20.53 | 24.78 | 22.42 | 20.70 |
|  | Lowest quintile | 27.63 | 29.87 | 20.37 | 27.22 | 22.65 | 24.91 |
| Caste | Scheduled caste | 13.23 | 13.06* | 19.23 | 19.11*** | 20.48 | 19.93* |
|  | Scheduled tribe | 8.00 | 9.85 | 9.50 | 7.02 | 7.86 | 10.12 |
|  | No caste | NA | NA | 1.06 | 2.93 | 2.59 | 2.71 |
|  | General caste | 78.77 | 77.09 | 70.22 | 70.93 | 69.08 | 67.24 |
| Religion | Hindu | 77.67 | 76.41* | 77.99 | 75.11*** | 77.78 | 73.53*** |
|  | Muslim | 15.87 | 17.41 | 16.01 | 21.07 | 16.68 | 21.40 |
|  | Christian | 2.08 | 2.11 | 2.19 | 1.83 | 1.88 | 2.18 |
|  | Sikh | 2.66 | 1.83 | 2.15 | 1.17 | 1.83 | 0.95 |
|  | Other/missing data | 1.73 | 2.24 | 1.67 | 0.83 | 1.83 | 1.93 |
| Type of residence | Urban | 22.69 | 22.24 | 23.11 | 17.93** | 23.33 | 35.24*** |
|  | Rural | 77.31 | 77.76 | 76.89 | 82.07 | 76.67 | 64.76 |
