# Supplementary material for: Is Economic Growth Associated with Reduction in Child Undernutrition in India?
Source: PLoS Med. 2011 Mar 8;8(3):e1000424. doi: 10.1371/journal.pmed.1000424 (PMC3050933; doi:10.1371/journal.pmed.1000424)
Supplement: Table S5 — State-wise proportion (%) of missing data for each survey year and the correlation of per capita state income (PCSI) and proportion of missing data in each state. (0.08 MB DOC) [file pmed.1000424.s005.doc]

**Table S5:** State-wise proportion (%) of missing data for each survey year; and the correlation of per capita state income (PCSI) and proportion of missing in each state.

|  | PCSI 93 | Proportion missing data - 1992-93 | | | PCSI 99 | Proportion missing data - 1998-1999 | | | PCSI 05 | Proportion missing data - 2005-2006 | | |
| --- | --- | --- | --- | --- | --- | --- | --- | --- | --- | --- | --- | --- |
| **State** |  | **Underweight** | **Stunting** | **Wasting** |  | **Underweight** | **Stunting** | **Wasting** |  | **Underweight** | **Stunting** | **Wasting** |
| Andhra Pradesh | 7006 | 9.27 |  |  | 15507 | 6.75 |  |  | 23755 | 17.59 |  |  |
| Assam | 5520 | 18.93 | 22 | 21.08 | 12282 | 17.66 | 30 | 30.58 | 17013 | 13.79 | 13.79 | 13.79 |
| Bihar | 4657 | 13.12 | 20.42 | 17.64 | 8600.5 | 11.99 | 19.92 | 19.87 | 12480 | 7.95 | 7.95 | 7.95 |
| Goa | 15332 | 8.2 | 10.5 | 11.82 | 42296 | 5.18 | 5.52 | 5.49 | 66135 | 23.84 | 23.84 | 23.84 |
| Gujarat | 9054 | 13.36 | 18.21 | 18.14 | 18864 | 10.19 | 13.55 | 13.57 | 29468 | 8.01 | 8.01 | 8.01 |
| Haryana | 10526 | 12.61 | 13.63 | 14.01 | 23121 | 4.49 | 9.47 | 9.36 | 37648 | 5.7 | 5.7 | 5.7 |
| Himachal Pradesh | 6896 | 4.7 |  |  | 20806 | 3.23 |  |  | 31139 | 9.51 |  |  |
| Jammu and Kashmir | 5400 | 17.64 | 20.07 | 22.29 | 13745 | 12.67 | 18.91 | 19.55 | 18630 | 13.54 | 13.54 | 13.54 |
| Karnataka | 7242 | 19.28 | 20.04 | 20.86 | 16603 | 10.73 | 12.06 | 12.82 | 23848 | 31.15 | 31.15 | 31.15 |
| Kerala | 6524 | 8.25 | 8.6 | 10.25 | 19294 | 7.64 | 15.25 | 14.98 | 27864 | 10.43 | 10.43 | 10.43 |
| Madhya Pradesh | 5516 | 14.88 |  |  | 12072.5 | 8.35 |  |  | 16272 | 7.71 |  |  |
| Maharashtra | 12010 | 17.7 | 19.13 | 20.69 | 23340 | 5.95 | 6.58 | 7.28 | 32979 | 25.87 | 25.87 | 25.87 |
| Manipur | 5929 | 13.47 | 15.71 | 16.96 | 13260 | 3.23 | 4.5 | 4.67 | 18386 | 9.83 | 9.83 | 9.83 |
| Meghalaya | 5934 | 29.34 | 40.47 | 40.04 | 14611 | 20.08 | 23.78 | 24.87 | 21915 | 42.28 | 42.28 | 42.28 |
| Mizoram | 7517 | 14.93 | 19.1 | 18.81 | 16443 | 6.76 | 8.85 | 8.67 | 22417 | 10.09 | 10.09 | 10.09 |
| Nagaland | 7730 | 23.7 | 27.51 | 28.51 | 13819 | 8.91 | 10.33 | 13.13 | 20998 | 15.83 | 15.83 | 15.83 |
| Orissa | 4662 | 20.62 | 23.18 | 23.61 | 10567 | 3.66 | 5.87 | 6.26 | 16306 | 7.6 | 7.6 | 7.6 |
| Punjab | 12934 | 13.17 | 13.17 | 14.92 | 25611 | 5.66 | 9.22 | 8.57 | 33158 | 6.85 | 6.85 | 6.85 |
| Rajasthan | 5315 | 21.78 | 28.47 | 29.53 | 13619 | 11.86 | 15.57 | 14.89 | 16515 | 8.51 | 8.51 | 8.51 |
| Tamil Nadu | 8051 | 21.47 |  |  | 19378 | 4.48 |  |  | 27137 | 13.27 |  |  |
| West Bengal | 6247 | 15.12 |  |  | 15826 | 5.25 |  |  | 22522 | 9.13 |  |  |
| Uttar Pradesh | 4794 | 14.47 | 18.47 | 17 | 11695.5 | 35.37 | 36.66 | 37.13 | 17058 | 17.97 | 17.97 | 17.97 |
| New Delhi | 17522 | 11.92 | 13.85 | 14.39 | 38682 | 15.35 | 19.78 | 21.07 | 55215 | 40.08 | 40.08 | 40.08 |
| Arunachal Pradesh | 8853 | 33.11 | 41.89 | 40.35 | 14107 | 5.88 | 7.64 | 9.43 | 22542 | 11.82 | 11.82 | 11.82 |
| Correlation with PCSI |  | -0.23 | -0.37 | -0.34 |  | -0.20 | -0.27 | -0.33 |  | 0.40 | 0.39 | 0.39 |
| p value of ***r*** |  | 0.28 | 0.12 | 0.15 |  | 0.36 | 0.26 | 0.17 |  | 0.06 | 0.10 | 0.10 |
