# Supplementary material for: Is Economic Growth Associated with Reduction in Child Undernutrition in India?
Source: PLoS Med. 2011 Mar 8;8(3):e1000424. doi: 10.1371/journal.pmed.1000424 (PMC3050933; doi:10.1371/journal.pmed.1000424)
Supplement: Table S6 — Odds ratios (95% confidence intervals) for associations between economic growth, sociodemographic factors, and indicators of undernutrition from logistic models that account for sampling weights. (0.07 MB DOC) [file pmed.1000424.s006.doc]

**Table S6:** Odds ratios (95% confidence intervals) for associations between economic growth, sociodemographic factors and indicators of undernutrition from logistic models that account for sampling weights.

|  |  | Underweight | Stunting | Wasting |
| --- | --- | --- | --- | --- |
| Characteristic | Category | Any | Any | Any |
| Economic growth |  | 1.01 (0.96, 1.06) | 1.11 (1.06, 1.17) | 0.96 (0.91, 1.02) |
| Survey year | 1992 | 1.00 | 1.00 | 1.00 |
|  | 1998 | 0.85 (0.76, 0.94) | 0.89 (0.79, 1.00) | 0.85 (0.74, 0.97) |
|  | 2005 | 0.67 (0.56, 0.80) | 0.53 (0.45, 0.64) | 1.02 (0.83, 1.26) |
| Age in months |  | 1.03 (1.03, 1.03) | 1.07 (1.07, 1.08) | 0.98 (0.97, 0.98) |
| Gender | Male | 1.00 | 1.00 | 1.00 |
|  | Female | 0.89 (0.86, 0.93) | 0.89 (0.85, 0.93) | 0.89 (0.84, 0.94) |
| Birth order | First | 1.00 | 1.00 | 1.00 |
|  | Second | 1.03 (0.98, 1.07) | 1.01 (0.97, 1.06) | 1.13 (1.07, 1.20) |
|  | Third | 1.04 (0.99, 1.10) | 1.00 (0.95, 1.05) | 1.16 (1.09, 1.25) |
|  | Fourth | 1.07 (1.00, 1.14) | 0.95 (0.89, 1.01) | 1.25 (1.15, 1.36) |
|  | Fifth and greater | 1.12 (1.05, 1.2) | 0.97 (0.90, 1.04) | 1.38 (1.26, 1.50) |
| Maternal age | 13 to 16 years | 1.27 (1.07, 1.51) | 1.23 (0.98, 1.55) | 1.01 (0.79, 1.28) |
|  | 17 to 19 years | 1.00 | 1.00 | 1.00 |
|  | 20 to 24 years | 0.91 (0.85, 0.98) | 0.90 (0.83, 0.98) | 1.09 (0.99, 1.20) |
|  | 25 to 29 years | 0.92 (0.85, 0.98) | 0.82 (0.76, 0.89) | 1.07 (0.97, 1.18) |
|  | 30 and more | 0.98 (0.91, 1.05) | 0.87 (0.80, 0.95) | 1.19 (1.07, 1.32) |
| Maternal education | No schooling | 1.98 (1.71, 2.30) | 2.13 (1.81, 2.50) | 1.27 (1.03, 1.55) |
|  | Primary | 1.78 (1.53, 2.07) | 1.90 (1.61, 2.24) | 1.23 (1.00, 1.50) |
|  | Secondary | 1.50 (1.30, 1.72) | 1.62 (1.39, 1.88) | 1.11 (0.92, 1.34) |
|  | College | 1.27 (1.08, 1.50) | 1.28 (1.08, 1.52) | 1.07 (0.86, 1.34) |
|  | >College | 1.00 | 1.00 | 1.00 |
| Paternal education | No schooling | 1.44 (1.31, 1.59) | 1.44 (1.29, 1.60) | 1.26 (1.10, 1.45) |
|  | Primary | 1.42 (1.29, 1.57) | 1.37 (1.22, 1.53) | 1.25 (1.09, 1.44) |
|  | Secondary | 1.27 (1.16, 1.38) | 1.28 (1.16, 1.41) | 1.19 (1.05, 1.34) |
|  | College | 1.15 (1.03, 1.27) | 1.13 (1.01, 1.26) | 1.08 (0.94, 1.24) |
|  | >College | 1.00 | 1.00 | 1.00 |
| Marital status | Married | 1.00 | 1.00 | 1.00 |
|  | No longer married | 0.92 (0.76, 1.11) | 1.04 (0.82, 1.32) | 0.93 (0.72, 1.21) |
| Household wealth | Highest quintile | 1.00 | 1.00 | 1.00 |
|  | Second quintile | 1.49 (1.38, 1.61) | 1.46 (1.34, 1.59) | 1.19 (1.07, 1.33) |
|  | Third quintile | 1.95 (1.79, 2.13) | 1.77 (1.61, 1.96) | 1.36 (1.21, 1.53) |
|  | Fourth quintile | 2.27 (2.07, 2.50) | 1.90 (1.70, 2.11) | 1.48 (1.31, 1.68) |
|  | Lowest quintile | 2.51 (2.27, 2.77) | 2.14 (1.91, 2.40) | 1.59 (1.39, 1.82) |
| Caste | General | 1.00 | 1.00 | 1.00 |
|  | Scheduled Caste | 1.18 (1.12, 1.25) | 1.20 (1.12, 1.29) | 1.12 (1.03, 1.20) |
|  | Scheduled Tribe | 1.24 (1.15, 1.34) | 1.11 (1.01, 1.22) | 1.36 (1.23, 1.50) |
|  | No caste | 1.09 (0.89, 1.33) | 1.02 (0.83, 1.25) | 1.10 (0.88, 1.38) |
| Religion | Hindu | 1.00 | 1.00 | 1.00 |
|  | Muslim | 1.04 (0.97, 1.11) | 1.04 (0.96, 1.12) | 0.98 (0.90, 1.07) |
|  | Christian | 0.83 (0.72, 0.97) | 0.83 (0.69, 0.99) | 0.84 (0.68, 1.03) |
|  | Sikh | 0.82 (0.70, 0.95) | 0.72 (0.61, 0.84) | 0.92 (0.77, 1.10) |
|  | Other | 1.01 (0.85, 1.20) | 1.14 (0.94, 1.39) | 1.05 (0.84, 1.29) |
| Residence | Urban | 1.00 | 1.00 | 1.00 |
|  | Rural | 0.93 (0.88, 0.99) | 0.93 (0.87, 1.00) | 0.99 (0.91, 1.07) |
